# A Simulated Intermediate State for Folding and Aggregation Provides Insights into ΔN6 β_2_-Microglobulin Amyloidogenic Behavior

**DOI:** 10.1371/journal.pcbi.1003606

**Published:** 2014-05-08

**Authors:** Sílvia G. Estácio, Heinrich Krobath, Diogo Vila-Viçosa, Miguel Machuqueiro, Eugene I. Shakhnovich, Patrícia F. N. Faísca

**Affiliations:** 1 Centro de Física da Matéria Condensada & Departamento de Física, Faculdade de Ciências, Universidade de Lisboa, Lisboa, Portugal; 2 Centro de Química e Bioquímica & Departamento de Química e Bioquímica, Faculdade de Ciências, Universidade de Lisboa, Lisboa, Portugal; 3 Department of Chemistry and Chemical Biology, Harvard University, Cambridge, Massachusetts, United States of America; Fudan University, China

## Abstract

A major component of *ex vivo* amyloid plaques of patients with dialysis-related amyloidosis (DRA) is a cleaved variant of β_2_-microglobulin (ΔN6) lacking the first six N-terminal residues. Here we perform a computational study on ΔN6, which provides clues to understand the amyloidogenicity of the full-length β_2_-microglobulin. Contrary to the wild-type form, ΔN6 is able to efficiently nucleate fibrillogenesis *in vitro* at physiological pH. This behavior is enhanced by a mild acidification of the medium such as that occurring in the synovial fluid of DRA patients. Results reported in this work, based on molecular simulations, indicate that deletion of the N-terminal hexapeptide triggers the formation of an intermediate state for folding and aggregation with an unstructured strand A and a native-like core. Strand A plays a pivotal role in aggregation by acting as a sticky hook in dimer assembly. This study further predicts that the detachment of strand A from the core is maximized at pH 6.2 resulting into higher aggregation efficiency. The structural mapping of the dimerization interface suggests that Tyr10, His13, Phe30 and His84 are hot-spot residues in ΔN6 amyloidogenesis.

## Author Summary

Dialysis-related amyloidosis (DRA) is a conformational disease that affects individuals undergoing long-term haemodialysis. In DRA the progressive accumulation of protein human β_2_-microglobulin (Hβ_2_m) in the osteoarticular system, followed by its assembly into amyloid fibrils, eventually leads to tissue erosion and destruction. Disclosing the aggregation mechanism of Hβ_2_m under physiologically relevant conditions represents a major challenge due to the inability of the protein to efficiently nucleate fibrillogenesis *in vitro* at physiological pH. On the other hand, ΔN6, a truncated variant of Hβ_2_m, which is also a major component of *ex vivo* amyloid deposits extracted from DRA patients, is able to efficiently form amyloid fibrils *de novo* in physiological conditions. This amyloidogenic behavior is dramatically enhanced in a slightly more acidic pH (6.2) compatible with the mild acidification that occurs in the synovial fluid of DRA patients. In this work, an innovative three-stage methodological approach, relying on an array of molecular simulations, spanning different levels of resolution is used to investigate the initial stage of the *de novo* aggregation mechanism of ΔN6 in a physiologically relevant pH range. We identify an intermediate state for folding and aggregation, whose potential to dimerize is enhanced at pH 6.2. Our results provide rationalizations for previous experimental observations and new insights into the molecular basis of DRA.

## Introduction

**Figure 1:**
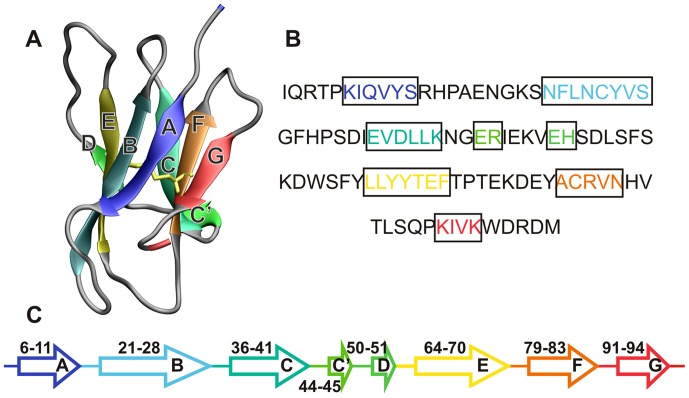
The wild-type human beta-2 microglobulin protein. The native structure of wild-type (WT) human beta-2 microglobulin (Hβ_2_m) (A), its primary sequence (B) and secondary structure content (C). Hβ_2_m comprises 99 residues arranged into a typical immunoglobulin (Ig) fold. It exhibits a sandwich-like structure formed by two sheets of anti-parallel β-strands. One of the sheets comprises strands A-B-E-D with the second sheet being formed by strands C-F-G. The native structure is stabilized by a disulfide bond (highlighted in yellow) established between residue Cys25 (located on strand B) and residue Cys80 (located on strand F). Another key structural feature of Hβ_2_m is the existence of a peptidyl-prolyl bond on the BC-loop (between His31 and Pro32), which adopts the thermodynamically unfavorable *cis*-conformation in the native structure. The location of each β-strand along the Hβ_2_m sequence is also shown (C). In the cleaved variant, ΔN6, the secondary structure assignment is similar with β-strands being defined in the following manner: 8–11(A), 21–27(B), 35–41(C), 44–45(C′), 64–70(E), 78–84(F), and 91–94(G).

β_2_-microglobulin (β_2_m) is a 99-residue protein with a typical immunoglobulin fold comprising seven anti-parallel β-strands stabilized by a disulfide bridge (**Fig. 1**) [1]. Upon dissociation from the MHC-I heavy chain, human β_2_m (Hβ_2_m) is catabolised in the kidneys. In individuals undergoing long-term hemodialysis the clearance process is strongly impaired and the levels of Hβ_2_m in the serum can increase up to 60-fold [2]. The progressive accumulation of Hβ_2_m in the osteoarticular system, presumably driven by its affinity for type-I collagen [3], eventually leads to amyloid assembly and the onset of dialysis-related amyloidosis (DRA), a pathological condition characterized by tissue erosion and destruction [4].

The wild-type Hβ_2_m (WT Hβ_2_m) does not form amyloid fibrils *in vitro* in the absence of *ex vivo* amyloid seeds [5], or additional factors such as Cu^2+^ [6], [7] or TFE [8]. This limitation makes the determination of the aggregation mechanism of Hβ_2_m in physiological conditions (37°C, pH 7.5) a particularly challenging conundrum. A major contribution towards its solution was the identification [9], [10], and atomic-level structural characterization [11], of an intermediate state (representing from 3.7±1.4% [10] up to ∼14±8% [9] of the equilibrium population) in the folding pathway of WT Hβ_2_m. The intermediate was termed I_T_ because its main structural trait is a non-native *trans* isomerization of Pro32. Enhanced fibrillogenesis in physiological conditions (including the ability to elongate and/or nucleate amyloid fibril assembly) has been observed in connection with an increase in the equilibrium concentration of I_T_ [10], [12], [13], indicating that I_T_ is highly amyloidogenic and a key player in Hβ_2_m fibrilogenesis. While relevance of I_T_ for β_2_m fibrillogenesis is widely acknowledged [9], [10], [14]–[16], alternative intermediate states, which are less native-like than I_T_, become relevant under different experimental conditions [8], [17]–[19]. Furthermore, a variety of environmental aspects have been found to directly affect the process of fibril formation by β_2_m including solubility, supersaturation and ultrasonication/agitation effects [20].

Recently, the single point mutant Asp76Asn (D76N), a naturally occurring variant of Hβ_2_m, was associated with the late onset of a fatal hereditary systemic amyloidosis characterized by extensive visceral amyloid deposits. However, and contrary to what occurs in DRA, in this newly discovered disease the plasma concentration of Hβ_2_m is not augmented [21], [22]. In vitro studies have shown that the Asp76Asn mutant is highly amyloidogenic, displaying an abundant (∼25%) equilibrium population of I_T_ under physiological conditions [22]. Another recently reported single point variant of β_2_m for which fibrillation occurs without seeding under physiological conditions is the Arg3Ala (R3A) mutant [23]. The latter, however, has not yet been associated with any conformational disorder.

In this work we focus on ΔN6, a truncated form of Hβ_2_m, lacking the first six N-terminal residues. This variant is potentially relevant because it represents ∼30% of *ex vivo* amyloid deposits extracted from DRA patients [24]. Radford and co-workers proposed that ΔN6 is a structural mimic of I_T_ because it populates a conformational state that reproduces the conformational features of I_T_ and represents 90% of ΔN6's in vitro equilibrium population [11]. While there is a broad agreement regarding the ability of ΔN6 to prime the fibrillar conversion of WT Hβ_2_m *in vitro* under physiological conditions, the mechanism by which it occurs is not consensual. In particular, Eichner and Radford proposed that monomeric ΔN6 conformationally converts WT Hβ_2_m into an amyloidogenic state in a mechanism akin to prion conversion [11], while Bellotti and coworkers challenged the prion-like hypothesis by reporting that the WT Hβ_2_m does not fibrillate with monomeric ΔN6 but rather with preassembled fibrils of ΔN6 [22]. However, and independently of these controversies, it is widely accepted that ΔN6 alone is able to efficiently nucleate fibrillogenesis in physiological conditions (t_lag_∼35 days, 80 mM) [11],[12],[25]. Furthermore, it displays an enhanced amyloidogenicity at pH 6.2 (t_lag_∼15 days, 80 mM) [11], i.e., in conditions compatible with the mildly-acidic character of the synovial fluid of DRA patients [26]. It has been suggested that the aggregation potential of ΔN6 stems from its unique ability to populate one or more aggregation-prone intermediate states [11]. Therefore, a complete picture of the aggregation mechanism of ΔN6 requires disclosing the process according to which it aggregates *de novo* starting from the self-association of aggregation-prone monomeric states. Addressing this challenge via molecular simulation is the major goal of the present work. By studying the early stage of aggregation of ΔN6 one expects to get insights into the amyloidogenicity of the full-length protein.

The large size of the system and the long timescales involved in the process of protein aggregation strongly restrict the use of classical molecular dynamics (e.g. based on the AMBER or GROMOS force fields) to explore it. For this reason researchers have been developing coarse-grained approaches to study protein aggregation [27]. One example is the symmetrised Gō potential used to study domain-swapping (DS) [28]. In DS two monomers exchange identical structural elements or “domains” to form a strongly bound dimer. Since the DS hypothesis is based on the association of two monomers into dimers, the mechanism of fibril formation from more than two identical proteins is still unclear. On the other hand since DS is a manifestation of concomitant folding and binding it requires the use of a simulation framework where the two processes compete directly with each other via a force field that accounts for competing intra- and intermolecular interactions [28]. Hβ_2_m fibrillogenesis has been reported to be initiated by dimerization of monomers [6], [12], [16], [29]–[31] including DS [32]. Here, however, we will not study the dimerization of Hβ_2_m that may result from DS. Instead, our goal in this study is to explore the early stage of the aggregation mechanism of the truncated variant ΔN6 that may occur as a side-effect of protein folding. More precisely, if aggregation-prone intermediate states (including highly native-like species) are populated along the folding pathway of ΔN6, they may start interacting with each other (e.g. via solvent-exposed hydrophobic residues) thus triggering the amyloid cascade. These aggregation-prone intermediates are a by-product of the folding process and likewise their formation is exclusively driven by intra-molecular interactions. Inter-molecular interactions will only start operating once the monomers representative of the intermediate state get within interaction range, which may eventually lead to their self-association into dimers. Our study seeks to explore this type of (*de novo*) aggregation route for the variant ΔN6 of Hβ_2_m by highlighting its topological aspects, i.e., the predictions reported here are strictly structure-based. In doing so, we use a three-stage computational protocol based on an array of tools as detailed in the Methods section. In the first stage, following our previous studies [33], [34], replica-exchange discrete molecular dynamics (RE-DMD) simulations of a full atomistic Gō model [35] are used to study the folding transition and to identify intermediate states in the folding pathway of ΔN6. The adopted level of structural resolution encompasses the effect on the folding mechanism of detailed atomic native contacts and fully takes into account side-chain packing, a fundamental ingredient of the folding process. Combined with RE-DMD simulations, the full atomistic Gō model enables equilibrium sampling of the conformational space, a task far beyond the possibilities of routinely used classical molecular dynamics protocols, especially for a system of the size of Hβ_2_m. While this simulation procedure captures the fundamental features of the folding process [36], it fails to include others. In particular, it neglects the effects of the pH, an important environmental parameter. Indeed, it is well known that changes in pH can induce conformational changes of varying degree, ranging from structural fluctuations to modifications in secondary structure content [37]–[39]. Furthermore, in the case of ΔN6 the pH turns out to be a particularly relevant parameter because – as stated before – the aggregation potential of this variant is remarkably sensitive to pH changes [11]. To identify the molecular roots of this dependence, it is thus crucial to establish how the pH affects the structure of the relevant conformational states, because structural changes at the monomer level (e.g. the reorganization of aromatic side chains, which are bulky and therefore natural players in the establishment of intermolecular interactions) will directly affect monomer association and ultimately dictate aggregation performance. In the second stage of our computational protocol, we investigate how the pH modulates an intermediate state's structure. Leaving aside its possible effect on large-scale conformational dynamics, we can afford to accurately capture the effect of pH by employing constant pH molecular dynamics (CpHMD) with explicit titration [37], [38], [40]–[43]. In doing so one also obtains conformations representative of the intermediate state with the most accurate representation of side-chain and backbone geometries, which is a requirement of the Monte Carlo ensemble docking (MC-ED) [33] protocol whose predictions depend critically on the structural accuracy of the analysed structures. The MC-ED is a low-resolution protocol that highlights the role of shape complementarity, a major driver of protein aggregation [44], [45]. It takes pairs of monomer conformations obtained with CpHMD to generate two ensembles of putative dimer structures (one obtained from monomers at equilibrated pH 6.2 and another at pH 7.2) where the number of residue pairs within interaction distance is maximized and the number of excluded volume interactions is minimized. The number of contacts thus evaluated provides a measure of the quality of the geometric matching between the two monomers. Therefore the MC-ED method allows predicting the residues that are most likely to trigger dimerization in an ensemble of dimers whose interface was optimized for shape complementarity. The MC-ED allows analyzing the association of an exceedingly large number of monomeric conformations while discriminating between the dimer structures that are prone to further oligomerize from those that are not. This is an important point because protein conformations are not static entities. Indeed, they have a dynamic nature leading to structural variability even for the native state. Thus, two pairs of self-associating conformations (representing the same conformational state) will not form exactly the same docking interface upon dimerization. By exhaustively docking thousands of equilibrated conformations collected from CpHMD simulations at pH 6.2 and 7.2 this work provides a probabilistic structurally resolved picture of the dimerization interface of the identified intermediate state, the native state of ΔN6 and also the native state of WT Hβ_2_m at physiological and near physiological pH. In doing so, it recapitulates and rationalizes previous experimental observations, and draws new insights into the aggregation mechanism of ΔN6, including the prediction of aggregation hot spots.

## Results

### Identification of an aggregation-prone folding intermediate

**Figure 2:**
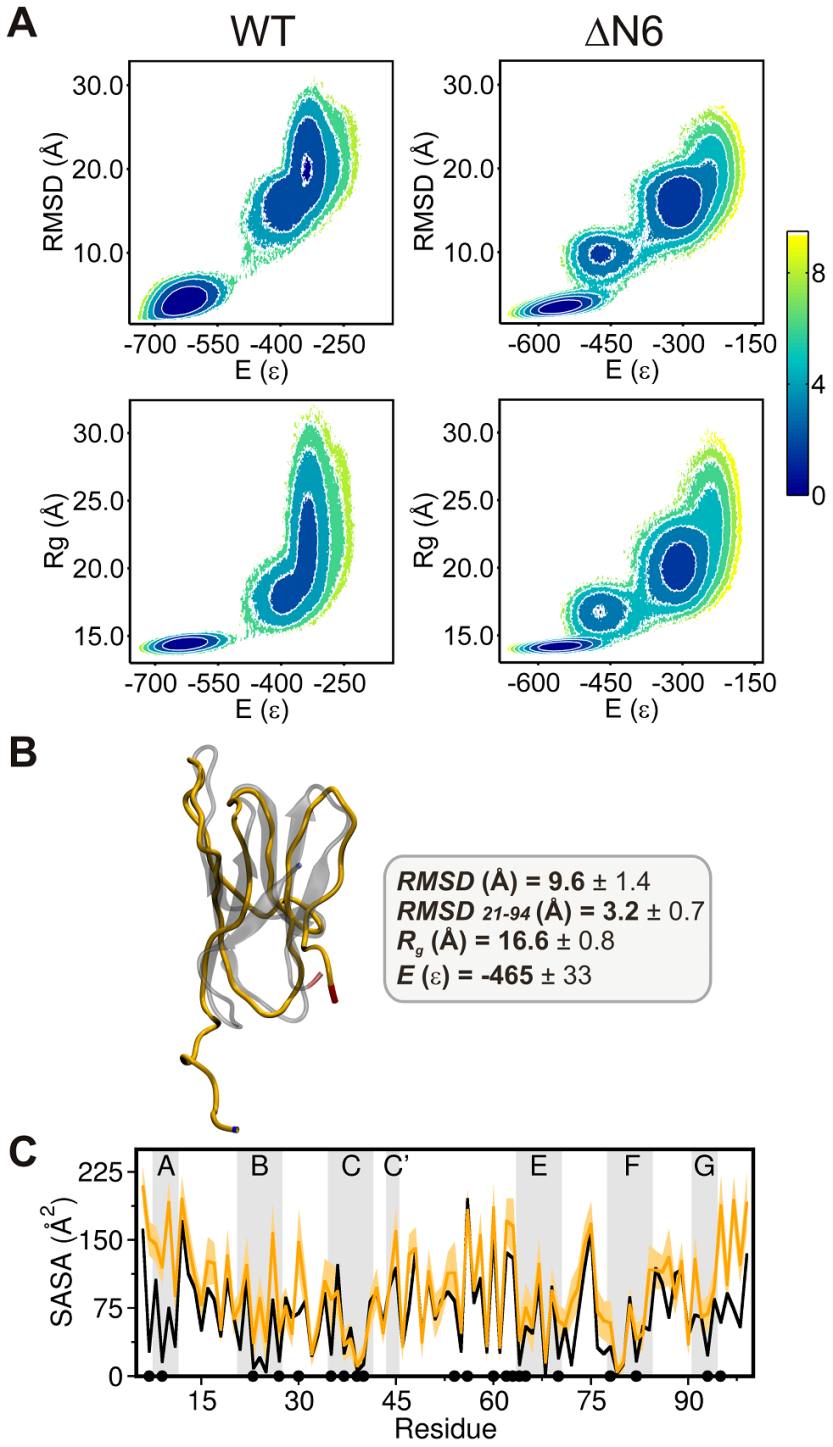
Characterization of the intermediate state populated by ΔN6. (A) Free energy surfaces for the Hβ_2_m and ΔN6 variant, showing an intermediate basin for the truncated mutant. The location of the free energy minima shows that the intermediate's energy, *E*, represents 83% of the native energy and its radius of gyration, *R*_g_, is 18% larger than that of the native state. The root-mean-square deviation, RMSD, measured with reference to the Hβ_2_m native structure is ∼10 Å. (B) Structure of a representative conformation (i.e. the conformation that is the closest to the cluster centroid) populated by the ΔN6 intermediate (which was isolated with structural clustering), and mean values (averaged over the intermediate's ensemble) of selected properties. The first Cα RMSD is measured for the whole chain taking as a reference the native structure (PDB ID: 2XKU). The second Cα RMSD_21–94_ was evaluated over the core region comprising residues 21 to 94 (i.e. strands B–G and connecting loops), after fitting to the core region of the native structure. This property highlights the conservation of this region in the intermediate species. (C) SASA values per residue were obtained as averages over the ensemble of intermediate conformations identified in the clustering and compared with those of the Hβ_2_m native structure (black line). The SASA values depicted were obtained with GROMACS v4.5.5 [80]–[82]. The dots represent the 21 hydrophobic core amino acids: Leu7, Val9, Leu23, Val27, Phe30, Ile35, Val37, Leu39, Leu40, Leu54, Phe56, Trp60, Phe62, Tyr63, Leu64, Leu65, Phe70, Tyr78, Val82, Val93, and Trp95. In the intermediate species 62% of the hydrophobic core residues have a noticeable increase in SASA.

The free energy (FE) surfaces at the folding temperature (*T_f_*), evaluated with the WHAM method [46], reveal a well-defined intermediate basin for ΔN6 that is not present in the FE surfaces of Hβ_2_m (**Fig. 2A**). The native and intermediate states populate ∼38% and 11% of the equilibrium ensemble at *T_f_*, respectively.

To isolate and structurally characterize the intermediate state populated by ΔN6, which we term ΔN6-I, we performed structural clustering over an ensemble of conformations collected from DMD simulations at fixed temperature (∼*T_f_*). The intermediate species preserves the *trans*-isomerization of Pro32 (as a consequence of the native-centric character of the Gō potential) and exhibits an unstructured/disordered strand A detached from a fairly conserved core region comprising residues 21 to 94 (i.e. strands B–G and connecting loops) (**Fig. 2B**). The detachment of strand A from the protein core and its structureless nature are likely the result of a smaller number of native interactions involving this secondary structural element, which decreases by 27% with regard to that observed in the full length species (**Fig. S5**). The evaluation of solvent accessible surface area (SASA) per residue reveals that 62% of the hydrophobic core residues become highly solvent-exposed in ΔN6-I with SASA exhibiting a 3- up to 7-fold increase in Leu7, Val9, Leu23 and Trp95, all located at the termini. Phe30 on the BC-loop, and Ile35 in strand C are also significantly more exposed to the solvent in the intermediate state (**Fig. 2C**). These observations are particularly relevant because the exposure of aggregation-prone hydrophobic patches has been pointed out as a hallmark of protein aggregation (reviewed in [47]) and suggest that the identified intermediate state has a high aggregation potential. We conjecture that strand A, by being exposed to the solvent, will be a particularly important structural motif for the early aggregation stage of ΔN6.

The Gō potential adopted in this work does not predict an equilibrium population of a similar full-length species, with a conserved core region and detached strand A, across the folding transition of the WT variant. However, there is experimental evidence that the amyloid-transition of the full-length Hβ_2_m is concomitant with a detachment of the N-terminal strand A [48] triggered by an acidic pH [19], [49]–[51] or Cu^2+^ binding [7], [48], [52]. Therefore it is likely that the full-length Hβ_2_m may undergo a similar conformational transition.

### Effect of pH on the structure of the intermediate state

The ΔN6-I intermediate state identified with the Gō model highlights the importance of native topology in determining the folding space. In order to investigate how the pH modulates the structure of the intermediate state, and, in particular, the degree of solvent-exposure of the unstructured strand A, we set up a series of CpHMD simulations that used the intermediate conformation as a topological template. This means that a structurally refined version of ΔN6-I was prepared by taking into account the structural information provided by the native-centric model. The refined structure was then used as the starting conformation in CpHMD simulations at pH 7.2 and pH 6.2.

The analysis of conformational ensembles taken from the equilibrated parts of CpHMD trajectories reveals that pH 6.2 has a striking effect on the region comprising strand A and the AB-loop. In ΔN6-I this region deviates significantly from its original position in the native structure as indicated by the large (mean) RMSD (16 Å) obtained after optimally superimposing each analyzed intermediate conformation over the native core region. The recorded RMSD at pH 6.2 represents an increase of 20% from that observed at pH 7.2 indicating a distinctively higher degree of solvent exposure at lower pH. On the other hand, the core region is better preserved at pH 6.2 with the (mean) RMSD decreasing up to 40% relative to pH 7.2 (**Table S1**).

The increased solvent-exposure of the N-terminal region (comprising residues 6 to 20) at pH 6.2 can be tentatively explained on the basis of a more favorable electrostatic contribution to the free energy of solvation at this pH. At a physiological/near-physiological pH Arg12 and Lys19 are permanently protonated, Glu16 is mostly deprotonated (only ∼0.3% protonation at pH 6.2), but the protonation state of His13 changes given the similarity between the medium pH and the average p*K*_a_ of its imidazole ring (6.0). Our data hints at the possibility of a direct connection between His13's protonation state and the SASA of the N-terminal region (comprising both strand A and the AB-loop) at pH 6.2 (**Fig. S1**). Therefore, one can argue that the higher degree of protonation of His13 at pH 6.2 leads to an increased solvent-exposure of that region which is concomitant with a favorably-enhanced electrostatic contribution to its free energy of solvation (it has been shown that protonation of the imidazole side chain produces a substantial increase of that histidine's absolute solvation free energy [53]).

### Dimerization propensity of the intermediate state

Seminal studies carried out by Miranker and co-workers have emphasized the importance of dimerization in the aggregation pathway of a mutational variant of Hβ_2_m (P32A) [6]. The latter is a structural mimic of an intermediate state (M*), which shares with I_T_ (and, therefore, with ΔN6) important structural features. Of note, a *trans* peptidyl-prolyl His31-Ala32 bond and the re-packing of several aromatic side chains within the hydrophobic core including Phe30 and Leu62. Furthermore, Eichner and Radford have shown that the M* intermediate elutes at a retention volume identical to that of I_T_ [12]. More recently, studies carried out by Radford and co-workers on the mutational variants P5G and ΔN6 (for which the intermediate I_T_ represents respectively ∼60% and 90% of the equilibrium ensemble in physiological conditions) showed that the first assembled oligomeric state in the amyloid pathway of both mutants is a dimer of I_T_ monomers [12]. Motivated by these findings, we carried out an exhaustive study of the dimerization interface in ΔN6 via MC-ED simulations.

We mapped the dimerization interfaces of the intermediate state (ΔN6-I) and of the native state (ΔN6-N) at pH 7.2 and 6.2 by docking pairs of conformations obtained from CpHMD simulations under those pH conditions. We also investigated the dimerization interface of the native state of WT Hβ_2_m (WT-N) as a control experiment.

**Figure 3:**
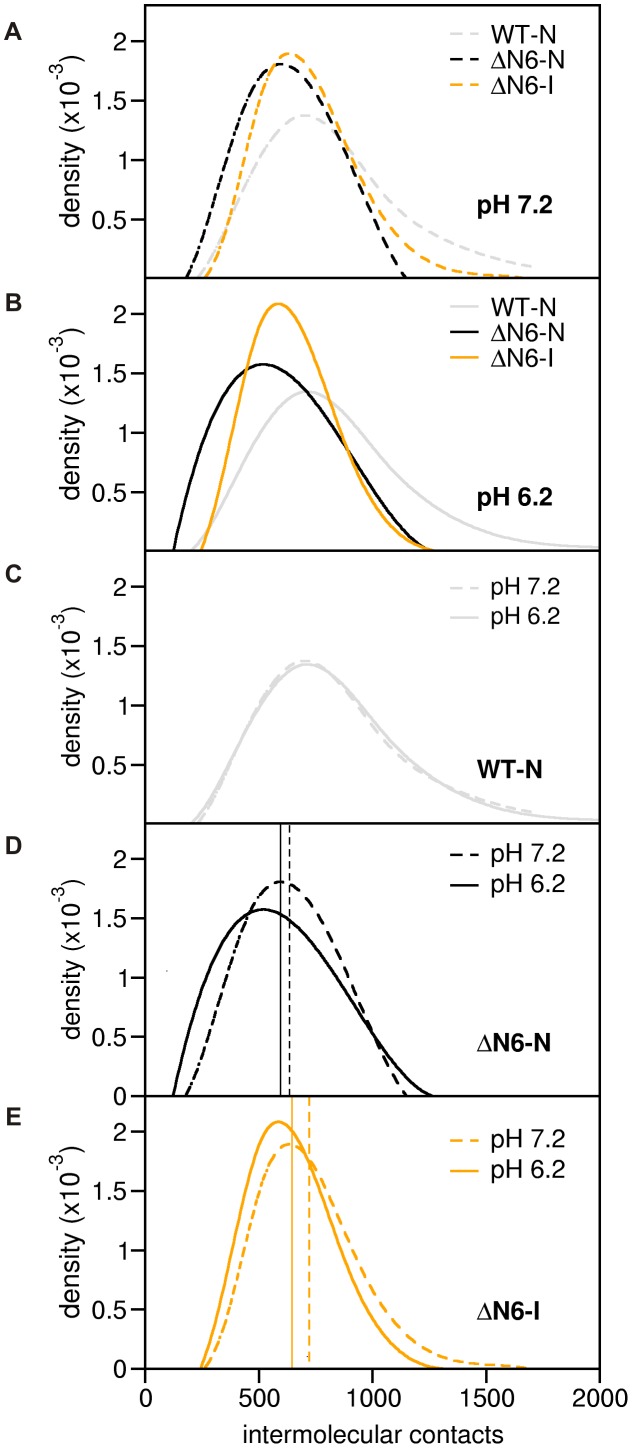
Intermolecular contact formation at pH 7.2 and 6.2. Density histograms for the number of intermolecular contacts established in dimers of WT-N, ΔN6-N and ΔN6-I at pH 7.2 (A) and pH 6.2 (B). To facilitate the comparison, the DHs for pH 7.2 and for pH 6.2 for WT-N (C), ΔN6-N (D), and ΔN6-I (E) are also shown. The vertical lines in the plots indicate the mean of the distributions.

In **Figure 3** we report density histograms (DH) of the number of intermolecular contacts at pH 7.2 (**Fig. 3A**) and pH 6.2 (**Fig. 3B**) for the dimers of the analyzed species. This property provides a quantitative measure of the quality of the geometric matching between the two monomers because each dimer conformation was optimized for maximum number of interactions and minimum number of excluded volume interactions. Each dimer interface was thus optimized for shape complementarity, a property that is considered a major driver of protein-protein association [44], [45]. In this sense the density histograms provide insight regarding the dimerization potential of each species. In the DHs the vertical lines indicate the mean, and the mode corresponds to the highest point of the distribution (representing the most probable number of intermolecular interactions in the population of dimers). In order to facilitate the comparison of these data, **Figures 3C–E** separately report the DHs for the two considered pH values. The analysis of the DHs reveals important findings. First, the high similarity between the curves obtained for the WT-N species suggests that it should conserve its dimerization propensity upon changing the pH from 7.2 to 6.2 (**Fig. 3C**). Since WT-N is the most populated state [9], [10], [15] of the *in vitro* equilibrium population in physiological conditions, our observation is consistent with the conservation of the aggregation behavior of Hβ_2_m across this pH range [25]. Our analysis further suggests that at the molecular level this behavior may be rooted in the conformational robustness exhibited by the monomeric form of WT-N across the analyzed pHs (**Table S2**), and, in particular, points out the importance of the protective role played by the N-terminus in maintaining the hydrophobic balance that stabilizes the native state [25]. Second, ΔN6-I forms dimers with number of intermolecular contacts given by the mode (mean) with a probability that is up to ∼52% (60%) higher than in the WT-N at pH 6.2 (for ΔN6-N this probability goes up to 51% at pH 7.2) (**Fig. 3D** and **Fig. 3E**). This is consistent with the higher amyloidogenicity of ΔN6 at physiological/near physiological pH. Third, when the pH decreases from 7.2 to 6.2, the mean and the mode decrease marginally for both ΔN6 conformational states. However, in the case of ΔN6-I, this mild decrease goes in tandem with a significant increase (up to 10%) in the probability of formation of the corresponding dimers (**Fig. 3E**). On the other hand, for ΔN6-N, the dimer conformations representative of the mean and mode are less probable at pH 6.2 than at pH 7.2 (**Fig. 3D**). Since it is likely that further oligomerization will be limited by nucleation of dimers, both measures (i.e. mean and mode) predict that ΔN6-I plays a major role in amyloid formation at pH 6.2.

### Structural mapping of the dimerization interfaces

**Figure 4:**
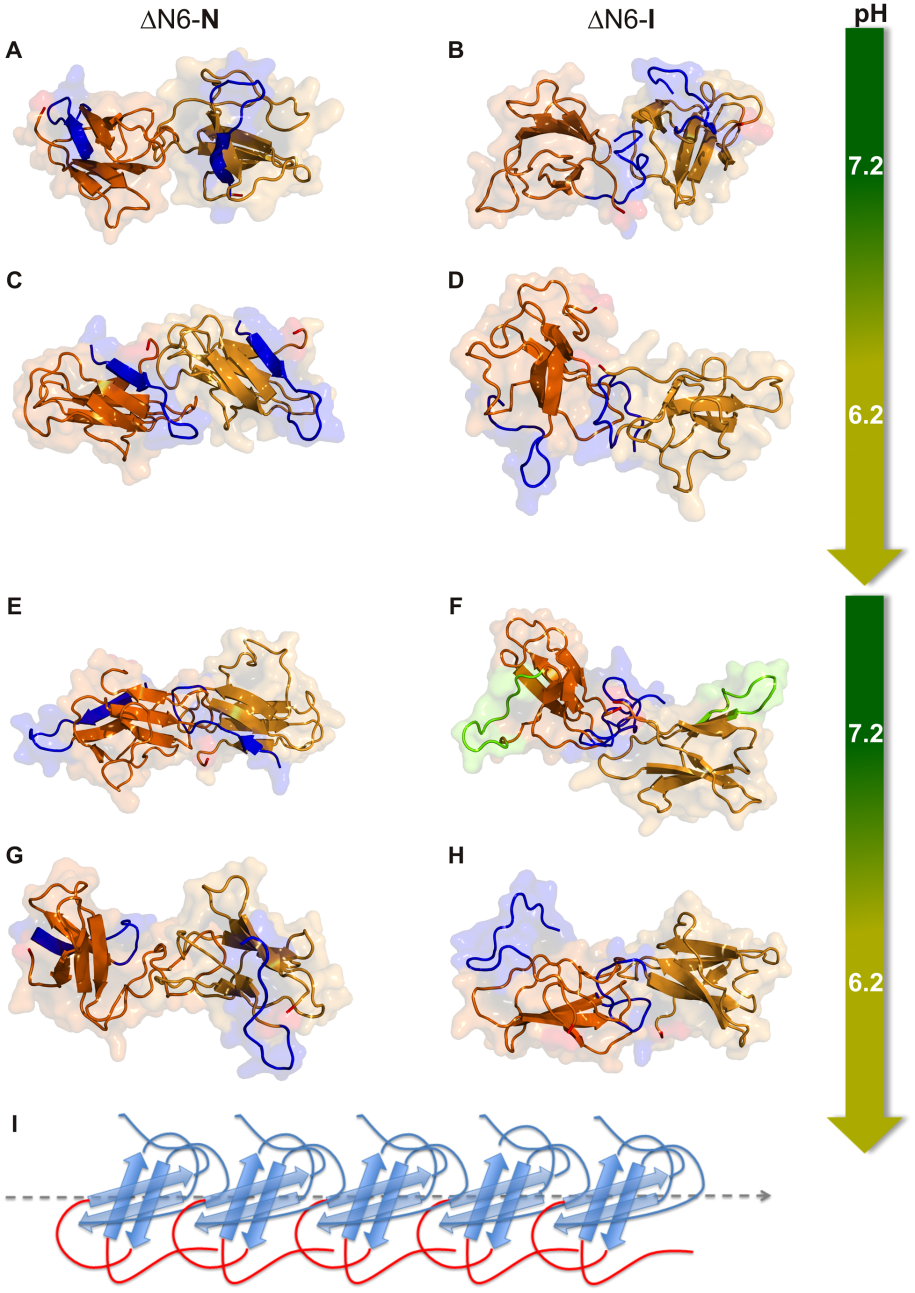
ΔN6-N and ΔN6-I dimer conformations. Representative dimer conformations with number of intermolecular contacts equal to the mode (mode dimers) and tail (strongly-matched dimers) of the density histograms. Mode dimers of ΔN6-N (A) and ΔN6-I (B) at pH 7.2, ΔN6-N (C) and ΔN6-I (D) at pH 6.2. Strongly-matched dimers of ΔN6-N (E) and ΔN6-I (F) at pH 7.2, ΔN6-N (G) and ΔN6-I (H) at pH 6.2. The ΔN6-I mode dimers (and the strongly-matched dimers at pH 6.2) highlight the involvement of strand A of one of the monomers in dimerization and the intrinsic possibility of further oligomerization via the second monomer's solvent-exposed strand A. If the unstructured strands A of both monomers are involved in dimerization (as in the strongly-packed dimer at pH 7.2, F) further growth is still possible via the highly solvent-exposed and aromatic-rich DE-loops (highlighted in green). In ΔN6-N the AB-loop and the “unstructured” DE-loop (which is detached from the protein's core and exposed to the solvent) account for the possibility of further oligomerization. Other examples of ΔN6-I ‘mode’ dimers are reported in **Fig. S3C** and **Fig. S3D**. (I) Pictorial representation of monomers of ΔN6-I that were packed ‘by hand’ to indicate a possible oligomerization pattern leading to amyloid fibrils.

In order to pinpoint the regions of the protein that are most likely to start dimerization, we have constructed probability contact maps for the dimer interfaces (**Fig. S2**). The probability of each intermolecular contact was evaluated by counting the number of times the contact is present in the ensemble of dimers that was used to determine the DH. We have also analyzed several representative dimer conformations (i.e. conformations with number of intermolecular contacts corresponding to the mode and tail of the DHs), to gauge their importance for further oligomerization (**Fig. 4** and **Fig. S3**). We have chosen to analyze the structure of ‘mode’ dimers for consistency reasons, i.e., because they exhibit the most likely number of intermolecular contacts (and a minimal number of excluded volume interactions) in the dimer interface, a property that quantifies the degree of geometric matching and shape complementarity of the interfaces in the ensemble of MC-ED generated dimers. On the other hand, the analysis of ‘tail’ dimers is particularly pertinent for the WT-N species because a unique feature of its DH is a rather extended tail indicating the formation of dimers with the strongest geometric matching (**Fig. 3C**). Since shape complementarity is a major driver of protein aggregation and is maximized for ‘tail’ dimers it is important to establish if/how the existence ‘tail’ dimers may affect the aggregation performance of Hβ_2_m.

We find that at both pH values dimerization of WT-N is majorly driven by the DE-loop (especially residues 56–60) (**Fig. S2A**). The analysis of several dimer conformations representative of the mode of the DH reveals that the most likely dimerization interface involves the DE-loop of one monomer that associates with the second monomer in several possible spots (**Fig. S3A**). On the other hand, the DE-loop directed interfaces of the strongly packed dimers are more specific, being based on loop-loop interactions involving the BC and DE aromatic-rich regions (**Fig. S3B**). Since the latter become unavailable for subsequent interaction, further oligomerization (via addition of another monomer) appears to be restricted to the potentially adhesive residues located on the EF-loop (e.g. Phe70 and Tyr78) and in the C-terminus (Trp95). Our analysis therefore predicts that the recruitment of the aromatic-rich regions in the WT-N best geometrically matched dimers' interfaces renders these dimeric entities particularly soluble thus lowering their aggregation potential (soluble dimerization was recently found to be a possible dead-end for aggregation in Ref. [54]).

In order to gauge the importance of residue 76 (located in the EF-loop) for the dimerization of Hβ_2_m we have selected a representative mode dimer of WT Hβ_2_m and used the program SCAP (included in the Jackal package [55]) to replace (in each monomer) the original amino acid Asp by an Asn thus mimicking the mutation that occurs in the systemic amyloidosis characterized by extensive visceral amyloid deposits. We then computed the electrostatic potential at the interface of both dimers (i.e. with and without the mutation) (**Fig. S4**). Our results are consistent with the enhanced amyloidogenicity observed *in vitro* for Asp76Asn with regard to Hβ_2_m [21], [22] because they indicate that the mutation contributes to stabilize the dimer by decreasing the amount of repulsive electrostatic interactions between the EF-loop of one monomer with the DE-loop of the second monomer.

In the case of ΔN6-N the pH has a modulating effect on the dimerization interface. First, the DE-loop is no longer the major player in dimerization, as both the AB- and BC-loops gain significant importance (**Fig. S2B**; **Fig. 4C** and **Fig. 4E**). The most important structural element for dimerization in ΔN6-I at pH 6.2 is the unstructured and detached strand A together with the adjoining AB-loop (**Fig. S2C**). Furthermore, inspection of several dimer conformations with number of intermolecular contacts equal to the mode (**Fig. 4B** and **Fig. 4D**; **Fig. S3C** and **Fig. S3D**) reveals that strand A facilitates fibril growth by imposing a rather straightforward oligomerization pattern. Indeed, strand A acts as a sticky ‘hook’ that recruits another monomer by interacting with its DE-, EF- or FG-loops (**Fig. 4B** and **Fig. 4D**; **Fig. S3C** and **Fig. S3D**) thereby leaving the second monomer's strand-A available for further growth. These ‘sticky hook’ interactions driven by strand A clearly drive a preferred oligomerization direction that could coincide with that of the amyloid fibril axis (**Fig. 4I**). Whenever monomer association involves strand A-strand A interactions, the resulting dimers can still grow via the BC- and DE-loops (**Fig. 4F** **; Fig. S3D**).

### Prediction of aggregation hot spots at different pH

In order to identify putative hot spots for aggregation, we computed the probability of intermolecular interaction per residue in the subset of the 50 most frequent intermolecular interactions. The latter were identified by taking the ensemble of dimers used in the evaluation of the corresponding DH. Pairs involving an aromatic amino acid and His or Lys dominate in ΔN6-I dimers at pH 6.2.

**Figure 5:**
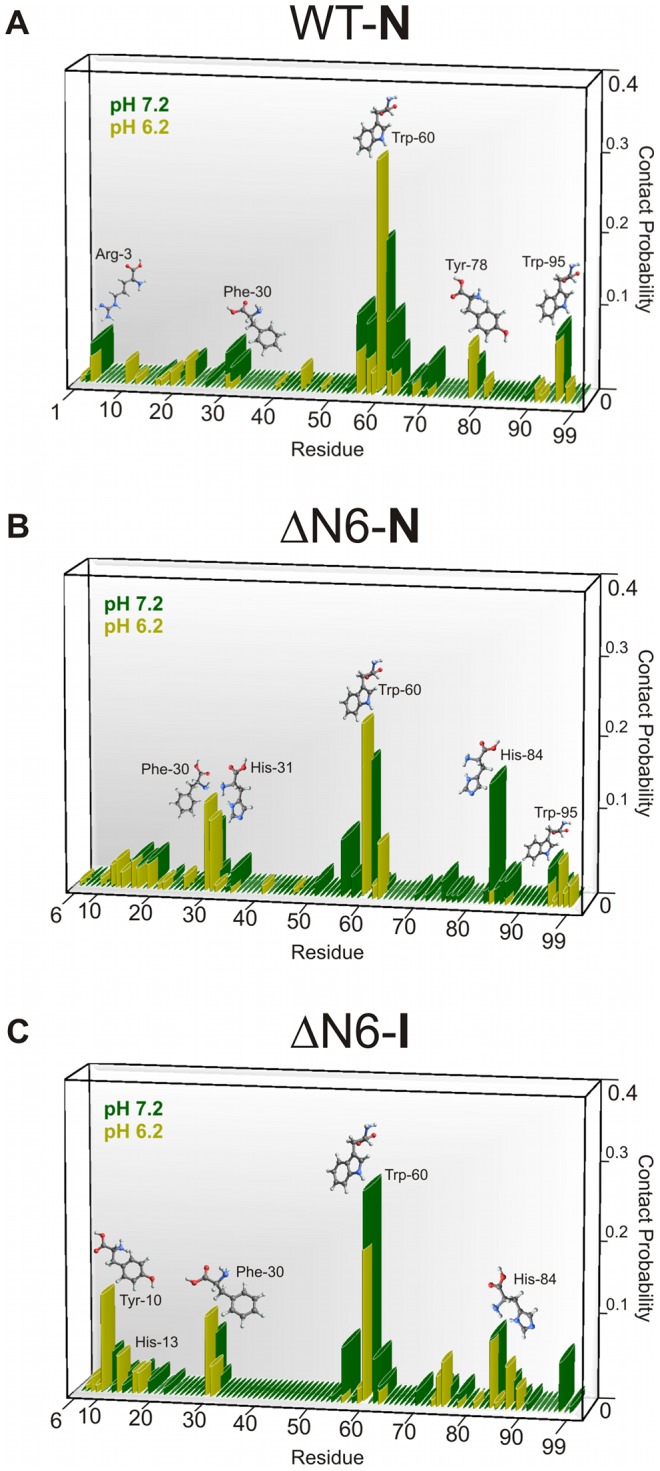
Putative aggregation hot spots. Intermolecular contact probability per residue evaluated over the ensemble of the 50 most frequent intermolecular contacts found in the dimers of WT-N (A), ΔN6-N (B) and ΔN6-I (C) at pH 7.2 and 6.2. The residues that exhibit a high probability to establish intermolecular contacts in ΔN6 (but not in WT-N) are considered to be putative aggregation hot spots.

In WT-N (**Fig. 5A**) the distinctive predominance of interactions involving the DE-loop illustrates this region's importance for dimerization. The relevance of the DE-loop in different experimental conditions has been acknowledged by several authors [11], [56], [57], including a recent study by Eisenberg and co-workers which reported a hinge motif in dimers of Hβ_2_m based on DE-loop swapping at pH 8 (in the presence of DTT) [32] and another study by Rennella *et al.* which reported the formation of Hβ_2_m dimers with a head-to-head arrangement of monomers driven by DE-loop interactions [16]. The aromatic residues Phe56, Trp60 (located on the DE loop's tip), Phe62, Tyr63 and the aliphatic Leu65 are expected dimerization spots because they assist the docking of Hβ_2_m onto the MHC-I heavy chain [2]. Phe62, Tyr63 and Leu65 were further shown to play an important role in fibril nucleation at acidic pH 2.5 [58]. The importance of Phe56 and Trp60 in β_2_m oligomer assembly based on D-D strand association (pH∼7) was reported in several studies [7], [57]. Of note, Trp60 was found to be the residue involved in the largest number of intermolecular contacts in Molecular Dynamics simulations that studied intermolecular interactions establishing between monomers of β_2_m [59]. The results reported here recapitulate that, with the exception of Leu65, DE-loop aromatic residues are important drivers of monomer association in Hβ_2_m. We further find that lowering the pH reduces the importance of residues Phe56, Lys58 and Phe62, while Trp60 becomes a particularly promiscuous interaction hub at pH 6.2 (**Fig. 5A**). However, the analysis of dimer's conformations whose formation is triggered by this region of the protein indicates that further oligomerization is not straightforward (**Fig. S3A** and **Fig. S3B**). In other words, our results suggest that while the DE-loop is certainly important for dimerization, the amyloid route that is triggered by this structural element is not the most efficient one. Since the native state is the dominant conformational state in physiological (and near physiological) pH, this observation rationalizes the little amyloidogenic character of WT Hβ_2_m in those conditions. Also, in line with this idea, we find that in ΔN6, which is considerably more amyloidogenic than Hβ_2_m, the importance of Trp60 (and nearby residues) is substantially reduced, especially in ΔN6-I at pH 6.2. This observation is particularly relevant because at this pH the cleaved mutant is more amyloidogenic.

In ΔN6-N (**Fig. 5B**), the region comprising the AB-loop (residues 10 to 20) exhibits an increased probability to form intermolecular contacts at both pH values. Due to increased solvent exposure of the BC-loop (residues 28 to 34) (**Fig. 2C**), there is also a significant enhancement of the participation of Phe30 and His31 (especially at pH 6.2). Direct involvement of the BC-loop in ΔN6 oligomer assembly (in physiological and near physiological pH) was reported in Ref. [11]. Furthermore, His31 was found to be a major contributor to the intermolecular contacts established in association interfaces within hexamers of H13F [7] and tetramers of DCIM50, the E50C Hβ_2_m mutant disulfide-linked homodimer [57], in physiological conditions (in the presence of Cu^2+^ or 20% TFE and fibril seeds, respectively). It was also observed to be a component of the non-covalent interface between two ΔN6 nanobody-trapped domain-swapped dimers (pH 5.0) in the respective crystal asymmetric unit along with the aromatic residues Phe56 and Trp60 [31]. The region comprising the end of strand F and the FG-loop (residues 84 to 90) – which is not involved in dimerization of Hβ_2_m – becomes especially relevant for ΔN6-N (pH 7.2) and ΔN6-I (at both pHs) (**Fig. 5C**). Interestingly, the stretch of amino acids ^83^N-^89^Q was implicated in the nanobody-driven domain-swapping aggregation of ΔN6 [31] and was shown to fibrillate into amyloid in a highly acidic pH 2.0 [60].

At neutral pH, both the BC- and DE-loops of ΔN6-N and ΔN6-I deviate significantly from their native positions (**Table S1** and **Table S3**). The cleaved N-terminus, which is more detached from the core in ΔN6-I, facilitates such conformational migration. Consequently, His84 located in the FG-loop (adjoined to the BC-loop), is more solvent exposed in ΔN6-N and ΔN6-I than in WT-N thus becoming an important interaction hub in the mutant's dimers (**Fig. 5B** and **Fig. 5C**). In the ΔN6-N dimer interfaces, His84 preferentially interacts with Phe56 and Trp60 at pH 7.2, while interaction with Tyr10 becomes relevant in ΔN6-I, especially at pH 6.2 (see next section for further details). The low amyloidogenicity of the Hβ_2_m mutational variant ΔN6/H84A in physiological or near-physiological pH [11] may thus reflect the absence of relevant interactions involving His84. Taken together, these findings thus point out to a direct participation of His84 in Hβ_2_m association, which adds up to a proposed indirect effect according to which His84 helps maintaining the *trans*-isomerization of Pro32 thus enhancing the population of I_T_ [61].

In ΔN6-I (**Fig. 5C**) residues Tyr10 and His13, located in strand A and start of the AB-loop, gain importance especially at pH 6.2. Previous studies reported the participation of these residues in association interfaces within hexamers of H13F [7] and tetramers of DCIM50 [57]. Furthermore, a single Tyr residue can act as the sole driving force triggering self-aggregation of a short polyalanine peptide (through cation - π and π-stacking interactions) [62]. His84 and Phe30 maintain their relevance for dimerization at both pHs. There is, however, a noticeable increase in the importance of Trp60 at pH 7.2 (in comparison with ΔN6-N). This happens because there is a larger migration of the DE-loop from the core region facilitating the participation of Trp60 in dimerization (**Table S1**). At pH 6.2 the EF-loop (residues 71–77), especially residues Glu74 and Lys75, also gains importance in dimer association (the EF-loop is not involved in dimerization in the WT-N). Overall, the most important feature of ΔN6-I dimerization is the striking increase in importance of strand A relative to WT-N.

### Fine-grained description of the dimerization interfaces

**Figure 6:**
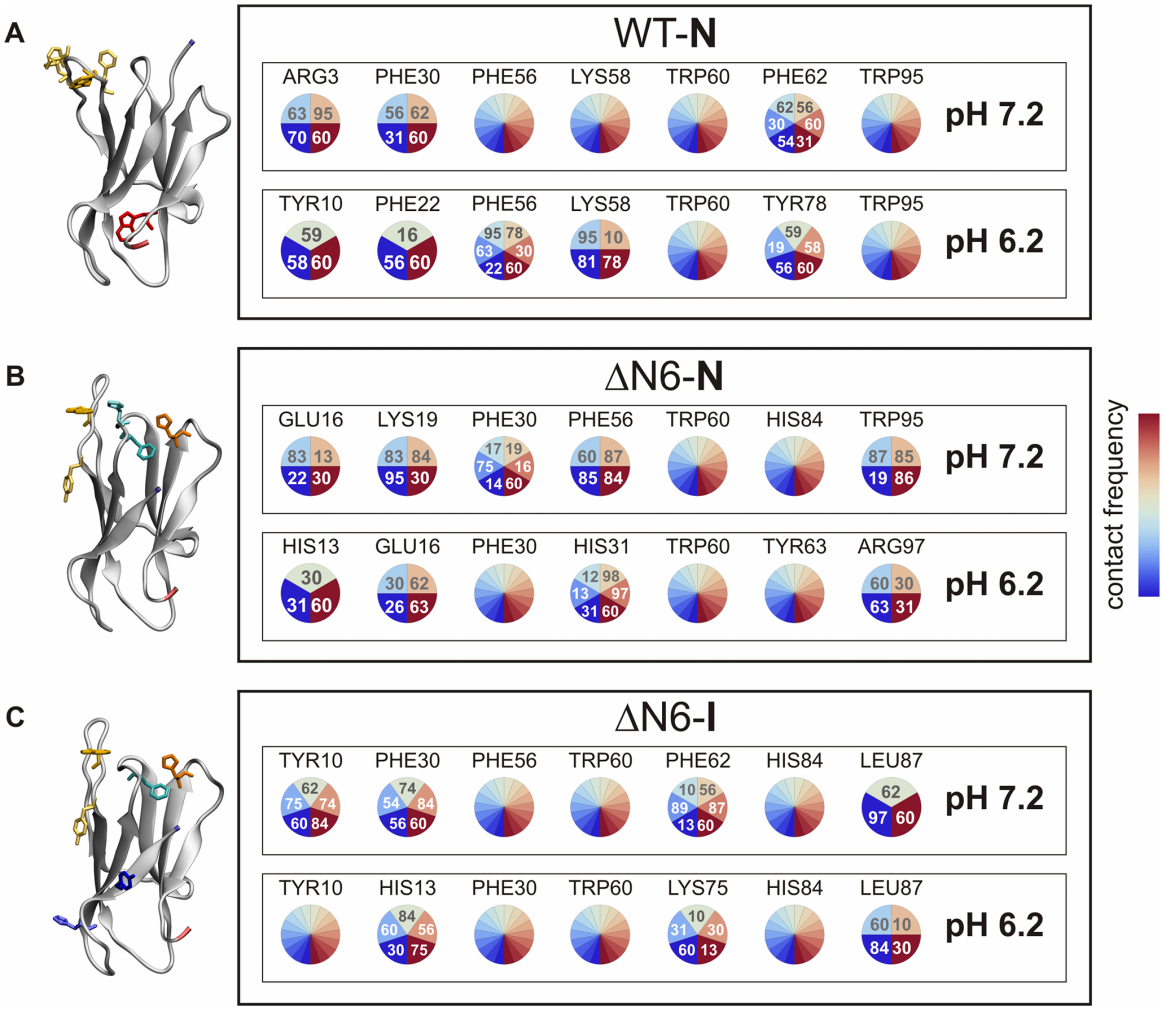
Interaction pies for the dimerization hot spots. Detailed analysis of the interaction partners of the dimerization hot spots (i.e. amino acids involved in the higher number of interactions within the set of the 50 most frequent intermolecular contacts). The numbers within each circle identify the residue (via its number along the protein sequence) that interacts with a putative hot spot, while the associated color represents the number of contacts found between the two-residues. The color code ranges from blue (i.e., small number of interactions) to red (i.e., large number of interactions). Whenever a hot-spot candidate has many interaction partners (more than six) a rainbow-like pie is used, and the residues are not explicitly identified. The hot-spots residues involved in the higher number of intermolecular interactions are mapped into the corresponding native structure (left column), and colored according to the color code adopted in **Figure 1**.

Here we identify the interaction partners of the predicted dimerization hot spots (**Fig. 6**) and pinpoint specific interactions (e.g. aromatic π-stacking, cation-π, and hydrophobic) that *may* contribute to efficiently stabilize the dimerization interface *in vitro* and *in vivo* (see **Table S4** and description therein of the 50 most frequent intermolecular contacts in ΔN6-I dimers at pH 6.2). Indeed, while the force field used in the MC-ED simulations does not explicitly take into account specific types of interactions, it is reasonable to determine if the predicted (structured-based) dimers meet the geometric requirements for the occurrence of such interactions (e.g. Cation-π interactions require that at least one of the atoms of the aromatic ring is located no further than ∼4.5 Å from one of the atoms carrying the net or partial positive charge – in His the positive charge can be located in the atoms Nδ1, Nε2, or Cε1 of the imidazole ring. In the present Gō model the maximum contact distance is ∼4.7 Å. Therefore, every contact between one His or Arg or Lys and one aromatic residue is within the Cation-π interaction distance).

#### Dimerization of WT-N

Phe56, Trp60, and Trp95 are the most prevalent amino acids in the WT-N interfaces at both pHs. These amino acids belong to two aromatic clusters (one formed by Phe30, Phe56, Phe62, Tyr63 and the other formed by Phe70, Tyr78, Trp95) of native Hβ_2_m. At neutral pH Phe56 interacts preferably with Tyr63 and Trp95, while Trp60 interacts mostly with Phe70 and Trp60. Trp95 interacts with several residues in the DE-loop. In a more acidic pH Phe56 interacts more with Phe30 and Trp60. The latter is also found in association with Trp95 and Phe62 with high probability. At pH 6.2 Tyr10 becomes a new, albeit less important, player in WT-N dimer interfaces interacting with Trp60 (possibly via aromatic π-stacking interactions), Asp59 and Lys58 (possibly via a stable cation-π interaction). Overall, there is a preponderance of intermolecular contacts involving aromatic amino acids within the DE-loop which interact preferably with other aromatic amino acids in the second monomer (**Fig. 6A**).

#### Dimerization of ΔN6-N

Phe30 and its neighboring residue His31 are putative hot-spots for ΔN6-N aggregation at both pHs. They interact preferably with Trp60, followed by Lys19 (Phe30) and Arg97 (His31). His84 becomes distinctively important in ΔN6-N dimers at pH 7.2 having as most probable interaction partner aromatic residues Phe56 and Trp60 (**Fig. 6B**).

#### Dimerization of ΔN6-I

Phe30 and His84 are potential hot spots for ΔN6-I aggregation, along with Tyr10 and His13, whose interaction potential increases substantially at pH 6.2. At this pH, Tyr10's most probable interaction partners include Trp60, His84, Glu74, and Asp59. His13 interacts preferably with Lys75, Phe56, His84 and Trp60, while His84 appears to prefer the aromatic residues Tyr10, Trp60, and Phe30, among others. His31 is also an important participant in ΔN6-I dimer interfaces at pH 6.2 where it establishes frequent interactions with Trp60, Lys75 and Tyr10. At pH 7.2, His13 and His31 have slightly fewer interaction partners but there is still a preferential association with Trp60, Phe56 (His13), and Tyr62 (His31) (**Fig. 6C**).

Residues His13, His31, and His84 are particularly interesting aggregation hot spots (at pH 7.2 and 6.2) in the dimerization of ΔN6 (with His13 assuming a more important role in the intermediate state and His31 in the native state at pH 6.2). These histidines interact preferentially with aromatic amino acids, including the highly promiscuous Trp60. Recently, the relevance of such associations for aggregation has been put forward in the case of islet amyloid polypeptides which were found to rapidly oligomerize into dimers and trimers via His-Tyr interactions [63]. Since the imidazole side chain of histidine has a p*K*_a_ of approximately 6.5 in solution [64] (in the current CpHMD simulations this particular p*K*_a_ takes values between 6.7<p*K*_a_<4), the protonation of histidines in the ΔN6 sequence has long been implicated with its increased amyloidogenicity at pH 6.2 [11]. A change of pH towards a more acidic value will favor the onset of strongly favorable cation-π interactions between the positively charged imidazole ring of His and the negatively-charged indole π-electron cloud in the aromatic amino acid. This “switch-like” behavior displayed by His-aromatic cation-π interactions, which is promoted by the protonation of His, produces an increase in the stability of the interaction in the order of 1 to 2 kcal mol^−1^ [65]. We conjecture that this effect should play an important role in ΔN6 dimerization producing a differentiated stabilization of its dimers at pH 6.2. This should be particularly relevant for the intermediate state dimers in which the occurrence of His-pairing interactions at pH 6.2 represents an increment of 23% relative to the neutral pH situation and for which the fraction of protonated His13/His31/His84 increases 3/26/1.25 times at pH 6.2. This increase in the fraction of protonated histidines upon a reduction of the pH from 7.2 to 6.2 is a direct consequence of the average p*K*_a_ values of the ΔN6-I histidines' imidazole rings. In fact, the average p*K*_a_ of the imidazole ring of His13/His31 in ΔN6-I is 6.0/5.2. The corresponding value for His84 could not be determined but it is low (<4) therefore explaining the lower increase in the fraction of protonated His84 at pH 6.2. The possibility/likelihood of cation-π interactions within ΔN6 dimer interfaces (**Table S4**) is compatible with their acknowledged relevance in a variety of protein-protein interfaces [66], [67]. Indeed, cation-π interactions have recently been shown to play an active role in molecular recognition in an intrinsically disordered oncoprotein family [68].

## Discussion

In recent years the identification and structural characterization of intermediate states for folding and aggregation [33], [69] has greatly contributed to a better understanding of the relation between the folding and aggregation landscapes [70]. The identification of these states in association with proteins of medical interest is of paramount importance. Indeed, not only it contributes to solve their aggregation mechanism but it also strengthens the need of including protein homeostasis as a therapeutic target for conformational diseases [71]. The work reported here illustrates how the combination of computational methods with different levels of resolution provides a unique opportunity to analyze the aggregation pathway and formulate testable predictions thus contributing to clarify the relation between folding and aggregation.

This study focused on the truncated mutant ΔN6 of protein Hβ_2_m and its dimerization mechanism. While the results reported here help gaining insights into the fibrillogenesis mechanism of the parent species, they do not entail an exclusive role of the truncated species in the actual fibrillogenesis pathway of the full-length protein.

Our study predicts the existence of an intermediate state for folding and aggregation in ΔN6. The intermediate preserves the *trans*-isomerization of Pro32 that characterizes I_T_ and a new structural trait: an unstructured strand A that detaches significantly from a fairly conserved core region comprising residues 21 to 94. The new intermediate state identified here represents a conformational excursion of the native state extending the loss of native structure already detected in the amyloidogenic intermediate I_T_ [10], [11]. The association of an unstructured/detached strand A with the onset of fibrillogenesis in β_2_-microglobulin was originally proposed by Verdone and co-workers [48], and subsequent studies have linked this structural trait with acidic pH [19], [49]–[51] or Cu^2+^ binding [7], [48], [52]. Therefore, it is likely that a similar conformational pathway may occur also with the full-length protein despite remaining undetected in the simulations carried out in this study. That this should the case is in fact demonstrated by the fibrillogenesis of the mutants D76N and R3A at neutral pH and without seeding. A lack of structure in one or both termini is a common feature shared by intermediates states that link the folding and aggregation landscapes [33], [69], [72].

Results reported here indicate that ΔN6 dimerizes with higher probability than WT Hβ_2_m, in line with its higher *in vitro* amyloidogenic potential and further predict that at pH 6.2 the intermediate state ΔN6-I identified in this work becomes the key player in ΔN6 dimerization. We find that the region comprising strand A and the AB-loop is critical for dimerization (especially at pH 6.2) and, presumably, to further oligomerization as well. Eichner and Radford reported a set of resonances in strand A and in the AB-loop of ΔN6 that shifted significantly at physiological pH depending on protein concentration, which is consistent with their involvement in aggregation. Interestingly, most of the chemical shifts of strand A are not defined because the residues resonate in a crowded region of the spectrum [11]. This may be taken as an indication of conformational liability for this part of the protein, making the NMR characterization of the proposed intermediate state a particularly challenging task.

The ΔN6(-N and I) dimers depicted in this work provide direct access to the atomic-level details associated to the participation of the N-terminal and BC-loop regions in Hβ_2_m oligomer assembly. In particular, our results reinforce the importance of the direct involvement of both regions in oligomerization which has been previously observed for several Hβ_2_m mutational variants usually in association with an enhanced amyloidogenicity [7], [11], [57].

The study of the dimerization interface we carried out for the WT form also recapitulates previous experimental findings. Namely, they reinforce the relevance of the DE-loop aromatic amino acids as important drivers of monomer association in Hβ_2_m. We found that monomer association driven by this region of the protein results into dimers of (WT) Hβ_2_m with a head-to-head arrangement of monomers that is similar to what is observed by other authors [16], [59]. The current work establishes that this (WT) Hβ_2_m mode of monomer association is such that further oligomerization is not straightforward. This is in agreement with the reported limited oligomerization of the native WT in physiological conditions [12], [56].

The comparative study of the dimerization interfaces we carried out for ΔN6 and WT Hβ_2_m allowed the prediction of putative dimerization hot spots in the truncated form. Residues Phe30, Phe56, Trp60 and Trp95 are universally important interaction hubs across the three species and pHs. They are able to establish a myriad of associations via their aromatic side-chains ranging from hydrophobic to the more stabilizing cation-π and π-stacking interactions. Trp60 is always highly promiscuous, Phe30 becomes distinctively important for ΔN6-N and ΔN6-I at pH 6.2, with Trp95 assuming a more important role in WT-N.

Finally, our results highlight the involvement of His84 in important interactions within ΔN6-N and ΔN6-I dimers therefore contributing to rationalize the low amyloidogenicity observed *in vitro* (at physiological and near-physiological pH) for the Hβ_2_m mutational variant ΔN6/H84A [11].

## Methods

**Figure 7:**
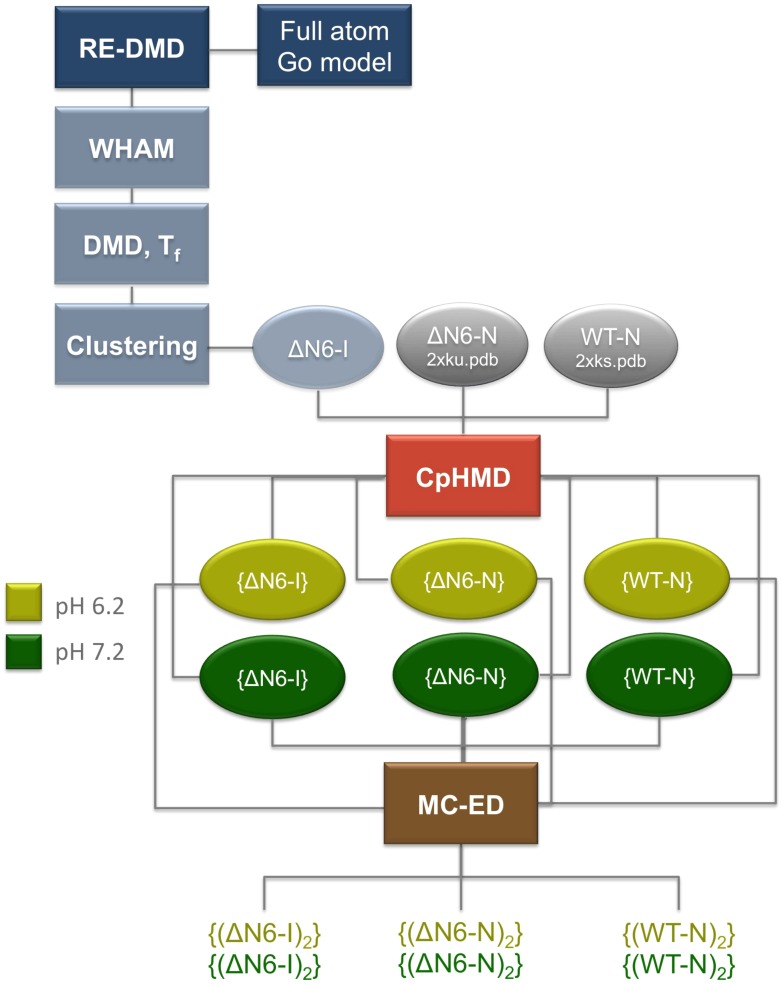
Methodological flow-chart. The methodological approach used in this work is based on a three-stage process. In the first stage, equilibrium sampling is performed with a full atomistic Gō model combined with replica-exchange discrete molecular dynamics simulations (RE-DMD). The transition temperature is calculated as the temperature at which the heat capacity peaks, and the heat capacity is computed from the mean square fluctuations in energy at each temperature considered in the RE simulations. The calculation of the free energy surfaces at the transition temperature – which indicates the existence of an intermediate state for ΔN6 – is done with the Weighted Histogram Analysis Method (WHAM). Structural clustering is performed over ensembles of conformations extracted from DMD simulations at the transition temperature to isolate the intermediate state populated by ΔN6 that is termed ΔN6-I. In the second stage, constant-pH molecular dynamics simulations (CpHMD) are used to study the effects of pH on the structure of ΔN6-I, native structure of ΔN6 (ΔN6-N) and native structure of the wild-type Hβ_2_m (WT-N). Two pH values are investigated, namely pH 6.2 and pH 7.2. The output of the CpHMD simulations consists of six ensembles of equilibrated conformations of ΔN6-I, ΔN6-N and WT-N at the two considered pH values. Finally, in the last stage of our approach, the conformations extracted from the CpHMD simulations are used as input for the Monte Carlo Ensemble Docking (MC-ES), which delivers ensembles of dimers of ΔN6-I, ΔN6-N and WT-N at the two considered pH values. The study of the dimerization interface is based on the statistical analysis of the ensembles of dimers.

This work employs different computational methodologies, described below. **Figure 7** shows a schematic representation of how these methods and their outputs are combined.

### Full atomistic Gō model

We consider a full atom representation where each non-hydrogen atom is taken as a hard sphere of unit mass. The atom's size is defined by scaling the relevant van der Waals (vdW) radius by a factor *α<1* [73]. Protein energetics is given by excluded volume interactions (which forbid hard-core clashes), bonded interactions, and non-bonded (or contact) interactions, all of which are all modeled by discontinuous, piecewise constant interaction potentials. Contact interactions are represented by a square-well potential whose depth is given by Gō energetic [35]. Thus, if atoms *i* and *j* are located in residues which are separated by more than two units of backbone distance the interaction parameter between them, *ε_ij_*, is given by
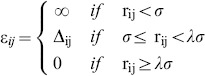
(1)


In the expression above *σ* = *α (r_0i_+r_0j_)* is the hard-core distance, *r_0i_* is the vdW radius of atom *i*, *λ* is a scaling factor that controls the range of attractive interactions, and *Δ_ij_* = −1ε (where ε is the energy unit) if *i* and *j* are in contact in the native conformation and is 0 otherwise. We followed Ref. [74] in treating the energetics of the disulfide bond in the same manner as we treat the other contact interactions. We set *α* = 0.80 and *λ* = 1.6 in order to have a well-behaved folding transition [73], [74]. This choice of parameters sets a cut-off distance of 4.7 Å (for methyl carbon), and leads to 957 native contacts in Hβ_2_m (PDB ID: 2XKS) and 899 native contacts in the ΔN6 mutant (PDB ID: 2XKU). The native contacts are distributed within the elements of secondary structure as reported in **Fig. S5**. The total energy of a conformation is computed as the sum over all atom pairs,
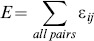
(2)


Further information about the adopted model can be found in Refs. [33], [34], [75]. Temperature is measured in units of ε/k_B_.

### Replica-exchange discrete molecular dynamics

The folding transition is explored with a discrete (or discontinuous) Molecular Dynamics (DMD) engine [76] and correct equilibrium sampling is achieved by using a standard replica-exchange (RE) Monte Carlo method [77] with a temperature grid that was calibrated to ensure a high acceptance probability (>90%) for the RE moves and replica ‘round-trips’ (i.e. moving from the top to the bottom of the temperature grid and back) with a mean cycle time of ∼50 RE moves. The equilibrated part of each simulation consisted of ∼5×10^10^ events per replica, and was used to collect uncorrelated data for the thermodynamic calculations. The folding (or melting) transition *T_f_* is usually estimated as the temperature at which the heat capacity *C_v_* attains its maximum value. Here, the *C_v_* is computed from the mean squared fluctuations in energy at each temperature considered in the RE simulations, in accordance with the definition 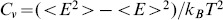. To compute the free energy as a function of different reaction coordinates (*E*, *R_g_*, RMSD) we have used the weighted histogram analysis method (WHAM) [46].

### Structural clustering

In order to isolate and structurally characterize the intermediate state populated by the ΔN6 truncated variant we started by running extensively long (up to 2.4×10^11^ events) DMD simulations at fixed temperature *T* (with *T* located within the transition region). A total number of three trajectories were considered. For each trajectory, a conformational ensemble (with up to 30k elements) was constructed by picking up equilibrated conformations (i.e. conformations sampled beyond the first folding transition). Subsequently, each conformational ensemble was analyzed with the *k*-means clustering algorithm of Brooks and co-workers as implemented in the MMTSB toolset [78]. The clustering radius cutoff was set to 9 Å (whenever the trajectories sampled both the native and intermediate basins) or 5–6 Å (if only the native basin was sampled).

### Constant-pH molecular dynamics

We performed CpHMD simulations at pH 7.2 and 6.2. The simulations of WT-N and ΔN6-N started from their NMR structures (PDB ID: 2XKS and 2XKU, respectively) and those of ΔN6-I started from five conformations that were built from the intermediate state obtained in DMD simulations. As reported previously (page 4 and **Fig. 2**) the intermediate state predicted by the full atomistic Gō model has two important structural features: it preserves the native core structure of ΔN6 (the RMSD of the region comprising strands B-G plus connecting loops to the same region in the native structure is 3 Å) and it exhibits a detached and unstructured strand A. To construct the starting conformations for the CpHMD we have firstly detached strand A from the core of the native conformation of ΔN6-N using PyMol (http://www.pymol.org) and subsequently relaxed these conformations via classical MD. All backbone dihedral angles modified were confirmed to be in Ramachandran allowed regions [79]. Relaxed conformations with an RMSD of 3 Å of the core region (measured to the core of the native structure) and five representative positions of strand A (that are consistent with those found in the ensemble of conformations representative of the intermediate basin) (see **Fig. 2A**) were then used as starting conformations for the CpHMD. By adopting this procedure one obtains conformations representative of the intermediate state with the most accurate/realistic representation of side-chain and backbone geometries, which is a requirement for the Monte Carlo ensemble docking protocol (see below) because the quality of the method's prediction depends critically on the structural accuracy of the analysed structures. We performed 30 simulations of 100 ns (3 systems, 2 pH values and 5 replicates). All simulations were performed using the stochastic titration constant-pH MD method implemented for the GROMACS package, developed by Baptista *et al.* [37], [38], [40]–[43]. The stochastic titration method consists essentially of a MM/MD simulation in which the protonation states of the protein are periodically replaced with new states sampled by Monte Carlo (MC) using Poisson-Boltzmann (PB) derived free energy terms. All His and acidic (Asp, Glu and C-ter) residues were titrated at all simulated pH values. Each constant-pH MD cycle was 2 ps long and the solvent relaxation step was 0.2 ps long. The MM/MD steps were performed using GROMACS 4.0.7 [80]–[82] and the GROMOS96 54A7 force field [83]. The leap-frog algorithm was used with a 2 fs time step. The structures were surrounded by 13641 SPC [84] water molecules in a rhombic dodecahedral box with periodic boundary conditions. The non-bonded interactions were treated using a twin-range cutoff of 8/14 Å and updating the neighbor lists every 10 fs. Electrostatic long range interactions were treated with a generalized reaction field [85] with a relative dielectric constant of 54 [86] and an ionic strength of 0.1 M [41]. The Berendsen coupling [87] was used to treat temperature (310 K) and pressure (1 bar) with coupling constants of 0.1 and 0.5, respectively. Solvent and solute were separately coupled to the temperature bath. Isothermal compressibility of 4.5×10^−5^ bar^−1^ was used. All bonds were constrained using the LINCS algorithm. The PB/MC calculations were done as previously described [88]. The MEAD 2.2.0 [89] software package was used for PB calculations. The atomic charges and radii [88] were taken from the GROMOS96 54A7 force field. A dielectric constant of 2 for the protein and 80 for the solvent were used. Grid spacing of 0.25, 1.0 and 2.0 Å were used in the finite difference focusing procedure [90]. The molecular surface was determined using a rolling probe of 1.4 Å and the Stern layer was 2 Å. The temperature was 310 K and the ionic strength was 0.1 M. The MC calculations were performed using the PETIT (version 1.5) [91] software with 10^5^ steps for each calculation. Each step consisted of a cycle of random choices of protonation state (including tautomeric forms) for all individual sites and for pairs of sites with a coupling above 2.0 p*K*_a_ units [91], [92], followed by the acceptance/rejection step according to Metropolis criterion [93]. Several tools from the GROMACS software package [80]–[82] were used for analysis and others were developed in-house. The DSSP criterion [94] was used to assign the secondary structure.

### Monte Carlo ensemble docking

The MC-ED method highlights the role of shape complementarity, which is a major driver of protein aggregation [44], [45]. The ultimate goal of the MC-ED [33] is to predict which parts of the protein are most likely to form geometrically matched protein-protein interfaces upon monomer self-association, and which residues may be critical for the onset of dimerization (i.e. dimerization hot-spots). This It is based on the assumption that any pair of monomers (representative of a specific conformational state, e.g., the intermediate state of ΔN6-I equilibrated at pH 6.2 or 7.2) may a priori dimerize should they come into interaction distance and on the importance of interface shape complementarity in protein-protein association. This assumption translates into building an ensemble of random pairs, over which the propensity to form geometrically matched interfaces will be analyzed statistically, as the starting point of the method. The random pairing introduces no bias and it is physically reasonable, since there is evidence that monomers approach each other via a long-range hydration force of enthalpic origin acting on the hydrophilic residues [95], before short-range, local hydrophobic interactions initiate dimerization and a well-packed interface may eventually be formed. The MC protocol employed to dock the two monomers represents protein conformations as rigid bodies and uses a series of random translations and rotations along the so-called docking axis (which is the axis that a priori guarantees a higher number of intermolecular contacts) combined with two cost functions that exclusively take into account packing interactions. For each pair of randomly selected conformations the MC returns an optimized docking interface with a maximum number of structural interactions (i.e. intermolecular contacts) and a minimum number of excluded volume interactions (i.e. atomic clashes). The detailed chemical structure of each amino acid is taken into account in the full atomistic protein representation and also to establish the intermolecular contact map (two amino acids are considered to be in contact in the dimer if any two atoms, whose size is given by the corresponding vdW radii, are within the interacting distance defined by the intramolecular Gō potential). To construct the density histograms the MC is applied consecutively to random pairs until the mean and the standard deviation of the number of intermolecular contacts converge. This typically amounts to dock up to 5000 pairs of conformations per studied species. The DHs are computed by counting (and normalizing) the number of dimers assigned to each bin of intermolecular contacts. The DHs provide a probabilistic description of the ensemble of random pairs from the point of view of the number of contacts of the geometrically matched interface each pair can form. They are used for comparison of dimerization propensity between species, and the ensemble of dimers that generates each DH is further used to identify the most likely structural parts of the protein which are key players in dimer formation, including the aggregation hot-spots. In this regard, since the heterogeneity in the amino acid interactions resulting from their different chemical nature is not taken into account into the corresponding cost function, the dimerization hot-spots will mostly depend on the quantity (and not on the quality) of intermolecular interactions established by each residue.

## Supporting Information

Figure S1 **Relation between the N-terminal region (β_A_+AB-loop) SASA and His13′ imidazole side-chain charge in the ΔN6-I dimers at pH 7.2 (green) and pH 6.2 (yellow).** Each point corresponds to an independent constant-pH MD trajectory mean SASA/His13 charge value. Error bars indicate the standard deviation in each trajectory. The correlation coefficients of the regressions have values of ∼0.6. The standard error of the regression coefficient at pH 7.2 (0.48) is, however, 3 times larger than the one obtained at pH 6.2 (0.15).
(PNG)

Figure S2 **Probability maps of the intermolecular contacts established in the (A) WT-N, (B) ΔN6-N, and (C) ΔN6-I dimer interfaces.** The location of each β-strand along the protein sequence is also shown for Hβ_2_m. In the case of the cleaved variant ΔN6, the secondary structure assignment is similar with β-strands being defined in the following manner: 8–11(A), 21–27(B), 35–41(C), 44–45(C′), 64–70(E), 78–84(F), and 91–94(G).
(PNG)

Figure S3 **WT-N and ΔN6-I dimers.** WT-N dimers (A–B) and ΔN6-I dimers (C–D). Loops are highlighted in green/cyan tones. In the ΔN6-I dimers the region comprising the A-strand and the AB-loop is highlighted in blue. At pH 6.2 the preferred association pattern in ΔN6-I mode dimers involves strand A of one monomer and the BC-, DE-, and/or EF-loop of the second monomer. The highly solvent-exposed strand A of the second monomer remains available for further oligomerization. At pH 7.2 strand A is not so critical for ΔN6-I dimer association, and the preferential association regions include the DE- and FG-loops.
(PNG)

Figure S4 **Surface electrostatic potentials of a typical (mode) WT-N dimer interface, with the original Asp76 (left) and the single-point mutation Asn76 (right), at physiological pH 7.2.** The color transitions from red – white – blue when going from negative (−5 k_B_T/e) – neutral (0 k_B_T/e) – positive (+5 k_B_T/e) electrostatic potential. The regions around residue 76, located in the EF-loop, are circled. In the WT-N dimer interfaces the EF-loop of one monomer interacts, almost exclusively through Tyr78, with the second monomer’ DE-loop (**Fig. S2A; Fig. S3A;** **Fig. 5A**). The dimer depicted is a representative example of such type of interaction. At physiological pH, the EF-loop of the WT-N has 3 negative charges and 1 positive charge. The DE loop displays 2 negative and 1 positive charges. In the WT-N dimer the interaction between both EF and DE loops can thus be affected by unfavorable electrostatic repulsions. The abundance of red color in the electrostatic map indicates a high load of negative charges in this type of interface (left) which is diminished when residue 76 is mutated into an Asn (right). The elimination of one negative charge from the EF-loop in the interface of the D76N dimer should therefore contribute to stabilize it, facilitating further oligomerization. The amino acids protonation states were attributed with PROPKA via the web server PDB2PQR v1.8 (http://nbcr-222.ucsd.edu/pdb2pqr_1.8/) and the calculation of the surface electrostatic potentials was done with the Adaptive Poisson-Boltzmann Solver – APBS v1.4 (http://www.poissonboltzmann.org/apbs/) and represented in VMD v1.8.7 (http://www.ks.uiuc.edu/Research/vmd/).
(PNG)

Figure S5 **Native contacts in the Gō model.** Number of native contacts per β-strand in the native structures of (WT) Hβ_2_m (PDB ID: 2XKS) and truncated variant ΔN6 (PDB ID: 2XKU). Secondary structure assignment is concurrent with the information provided in the PDB data files. In the WT form β-strands are defined within the sequence segments 6–11(A), 21–28(B), 36–41(C), 44–45(C′), 50–51(D), 64–70(E), 79–83(F), and 91–94(G). In the cleaved variant β-strands are defined in the following manner: 8–11(A), 21–27(B), 35–41(C), 44–45(C′), 64–70(E), 78–84(F), and 91–94(G).
(PNG)

Table S1 **Structural characterization of the monomeric ΔN6-I sampled in the CpHMD simulations.** The second column displays the mean Cα RMSD of the full chain fit to the native structure (PDB ID: 2XKU). The fifth column displays the SASA of the (β_A_+AB-loop) region. The remaining columns display the mean Cα RMSD of selected protein regions after fitting the core region, which comprises residues 21 to 94 (i.e. strands B–G and connecting loops), to the native structure. The RMSD of the (β_A_+AB-loop) region was obtained by taking into account the residues belonging to those structural elements plus the remaining N-terminus residues (residues 6–20). The BC region comprises residues 21–41 (strands B–C and BC-loop), the DE-region residues 50–70 (strands “D”–E and DE-loop) and the FG-region residues 78–94 (strands F–G and FG-loop). Averages were obtained from ensembles with ∼5000 conformations.
(DOC)

Table S2 **Structural characterization of the monomeric WT-N sampled in the CpHMD simulations.** The second column reports the mean Cα RMSD of the full protein chain fit to the native structure (PDB ID: 2XKS). Values for the mean Cα RMSD_21–94_ were evaluated over the core region comprising residues 21 to 94 (i.e. strands B–G and connecting loops), after fitting to the core region of the native structure. Averages were obtained from ensembles with 2883 (6.2) and 3003 (7.2) conformations.
(DOC)

Table S3 **Structural characterization of the monomeric ΔN6-N sampled in the CpHMD simulations.** The second column displays the average Cα RMSD of the full chain fit to the native structure (PDB ID: 2XKU). The remaining columns display the average Cα RMSD of selected protein regions after fitting the core region, which comprises residues 21 to 94 (i.e. strands B–G and connecting loops), to the native structure. The RMSD of the (β_A_+AB-loop) region was evaluated by taking into account the residues located within those structural elements plus the N-terminus residues (residues 6–20). The BC region comprises residues 21–41 (strands B–C and BC-loop), the DE-region residues 50–70 (strands “D”–E and DE-loop) and the FG-region residues 78–94 (strands F–G and FG-loop). Averages were obtained from ensembles with 1902(6.2) and 3003 (7.2) conformations.
(DOC)

Table S4 **50 most probable intermolecular contacts in ΔN6-I at pH 6.2.** The color code is as follows: aromatic amino acids (i.e., Phe, Tyr, or Trp) interacting with Lys, Arg or His are highlighted in light blue. Aromatic amino acids interacting with an aromatic counterpart are highlighted in red (aromatic amino acids pairs can interact through their aromatic π rings in π–stacking interactions) while hydrophobic pairs (with an aromatic or aliphatic side chain) are highlighted in orange. Amino acids with electrically charged side chains (acidic – Asp and Glu; basic – Arg, Lys, and His) are highlighted in blue. Pairs involving an aromatic amino acid and His or Lys dominate the top 50 most frequent intermolecular contacts in ΔN6-I dimers at pH 6.2. These pairs can interact through cation-π interactions involving the aromatic π-ring and the positively charged moiety on Lys or protonated His (when neutral, His can establish aromatic–aromatic or π–stacking interactions with its aromatic partner as well as δ^+^-π interactions due to polarization). Arg and Lys side chains are protonated (positively charged) in the range of pHs studied (p*K*_a_>>pH). Glu and Asp RCOO^−^ side chains are mostly unprotonated (negatively charged) in physiological or near-physiological pHs. His imidazole ring can become protonated in the 6.2–7.2 pH range. Assuming an average p*K*_a_ of the imidazole ring side chain of approximately 6.5, only 17% of all His in the system become protonated at a pH of 7.2 while at a slightly lower pH of 6.2 this value increases up to 67% (the side chain p*K*_a_ of a buried His can assume a value under 6.5 thus lowering the pH for which it becomes protonated).
(DOC)
