# Supplementary figures and images for: A Simulated Intermediate State for Folding and Aggregation Provides Insights into ΔN6 β2-Microglobulin Amyloidogenic Behavior
Source: PLoS Comput Biol. 2014 May 8;10(5):e1003606. doi: 10.1371/journal.pcbi.1003606 (PMC4014404; doi:10.1371/journal.pcbi.1003606)

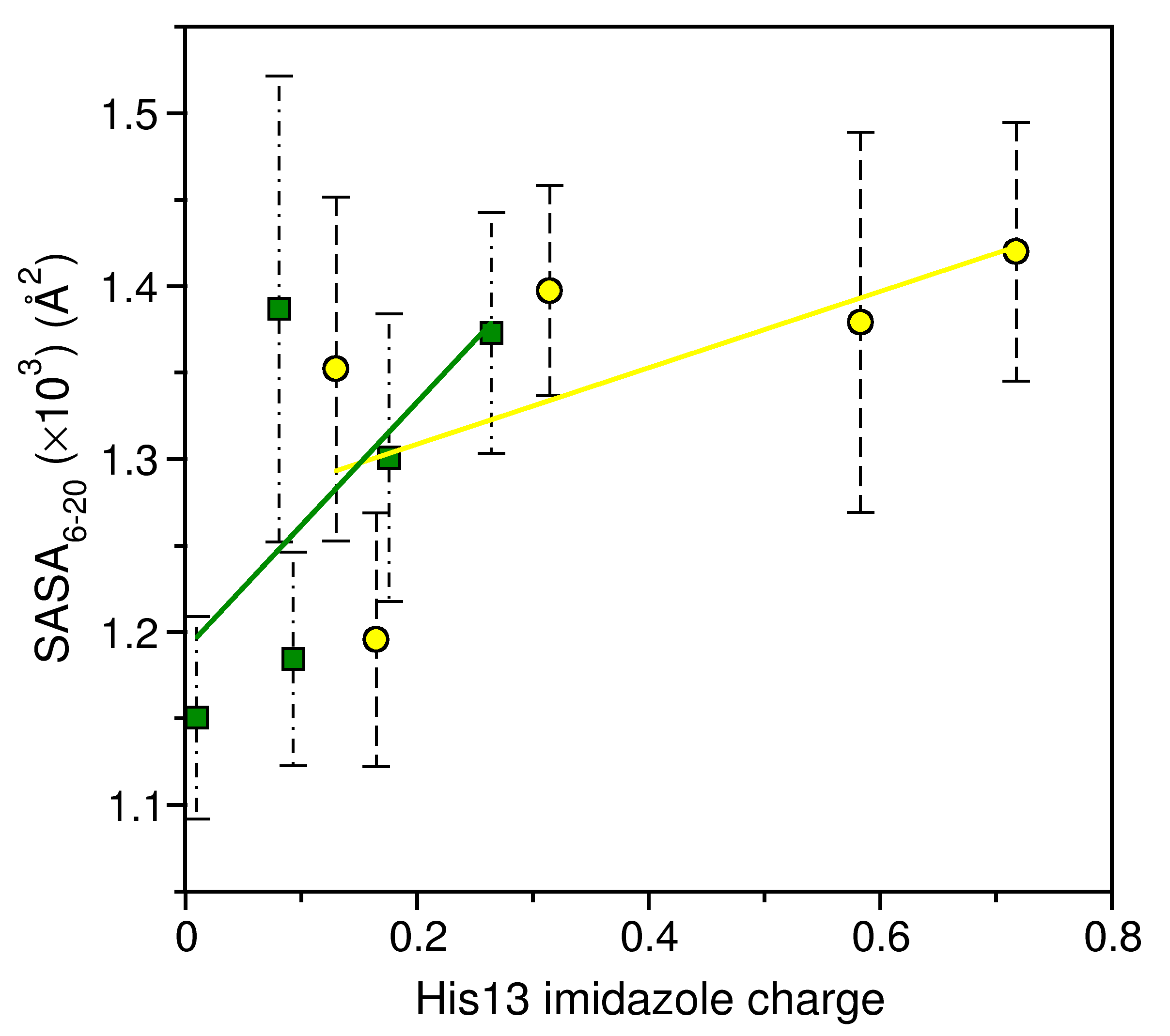

Supplement: Figure S1 — Relation between the N-terminal region (βA+AB-loop) SASA and His13′ imidazole side-chain charge in the ΔN6-I dimers at pH 7.2 (green) and pH 6.2 (yellow). Each point corresponds to an independent constant-pH MD trajectory mean SASA/His13 charge value. Error bars indicate the standard deviation in each trajectory. The correlation coefficients of the regressions have values of ∼0.6. The standard error of the regression coefficient at pH 7.2 (0.48) is, however, 3 times larger than the one obtained at pH 6.2 (0.15). (PNG) [file pcbi.1003606.s001.png]

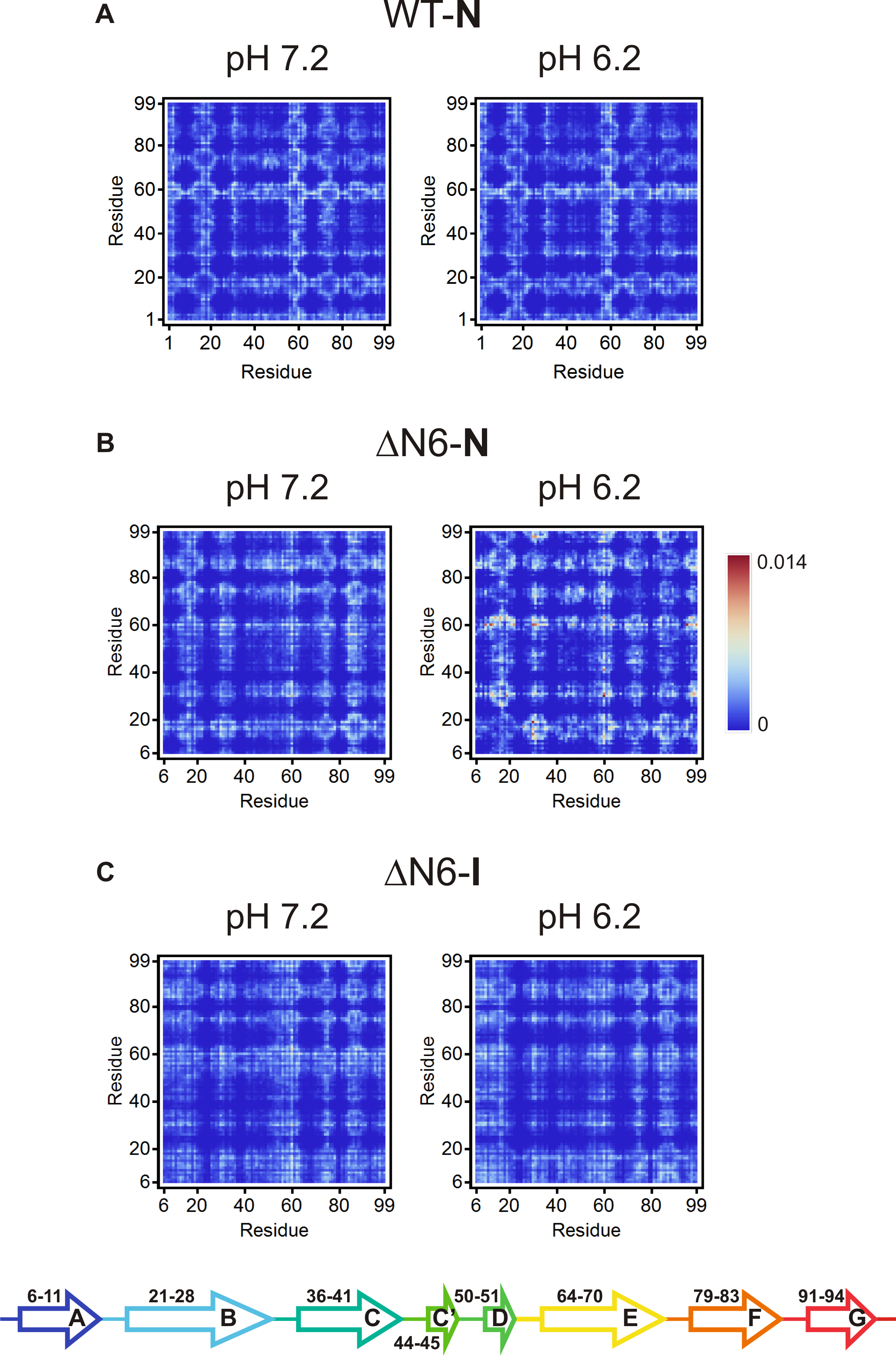

Supplement: Figure S2 — Probability maps of the intermolecular contacts established in the (A) WT-N, (B) ΔN6-N, and (C) ΔN6-I dimer interfaces. The location of each β-strand along the protein sequence is also shown for Hβ2m. In the case of the cleaved variant ΔN6, the secondary structure assignment is similar with β-strands being defined in the following manner: 8–11(A), 21–27(B), 35–41(C), 44–45(C′), 64–70(E), 78–84(F), and 91–94(G). (PNG) [file pcbi.1003606.s002.png]

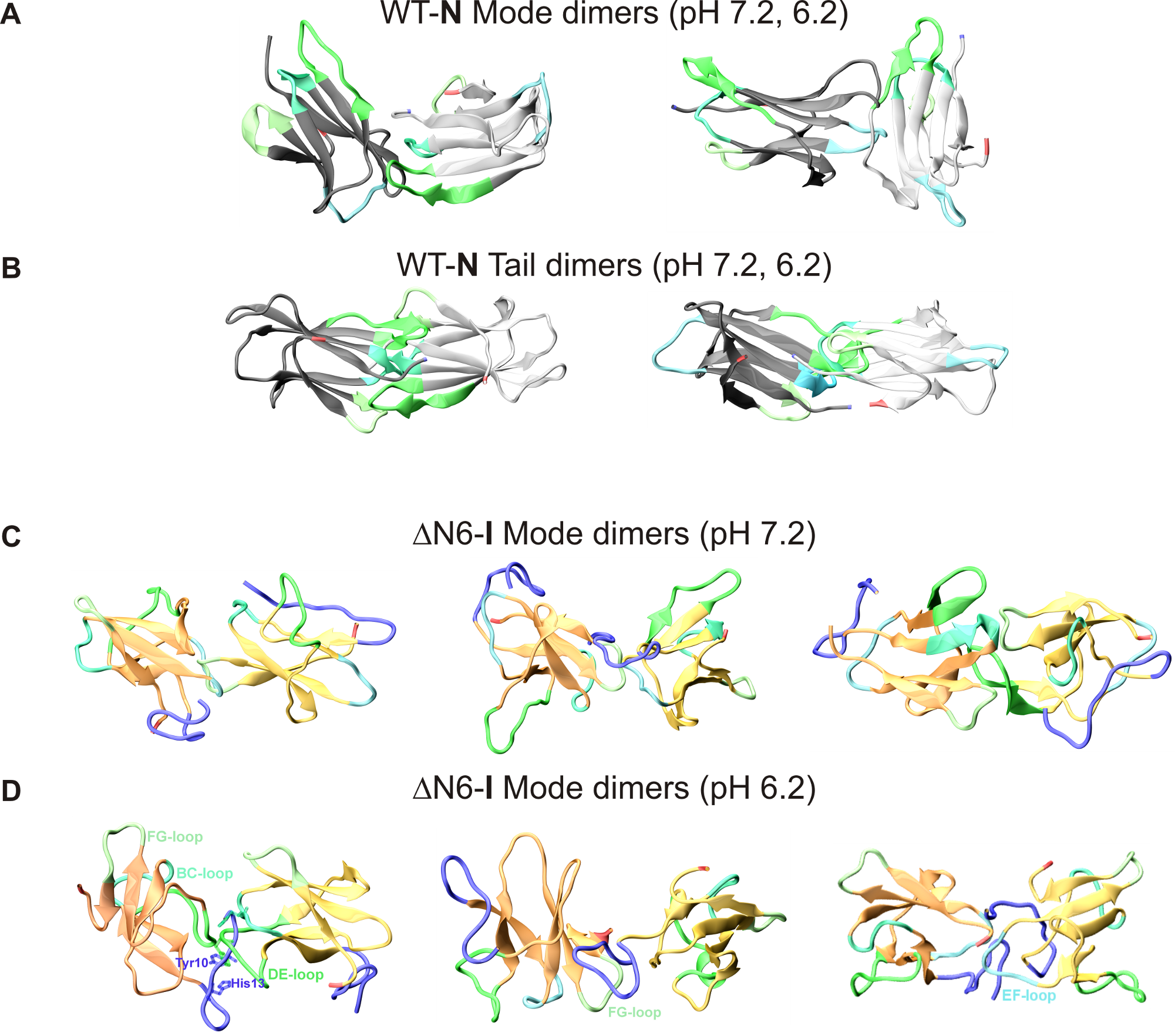

Supplement: Figure S3 — WT-N and ΔN6-I dimers. WT-N dimers (A–B) and ΔN6-I dimers (C–D). Loops are highlighted in green/cyan tones. In the ΔN6-I dimers the region comprising the A-strand and the AB-loop is highlighted in blue. At pH 6.2 the preferred association pattern in ΔN6-I mode dimers involves strand A of one monomer and the BC-, DE-, and/or EF-loop of the second monomer. The highly solvent-exposed strand A of the second monomer remains available for further oligomerization. At pH 7.2 strand A is not so critical for ΔN6-I dimer association, and the preferential association regions include the DE- and FG-loops. (PNG) [file pcbi.1003606.s003.png]

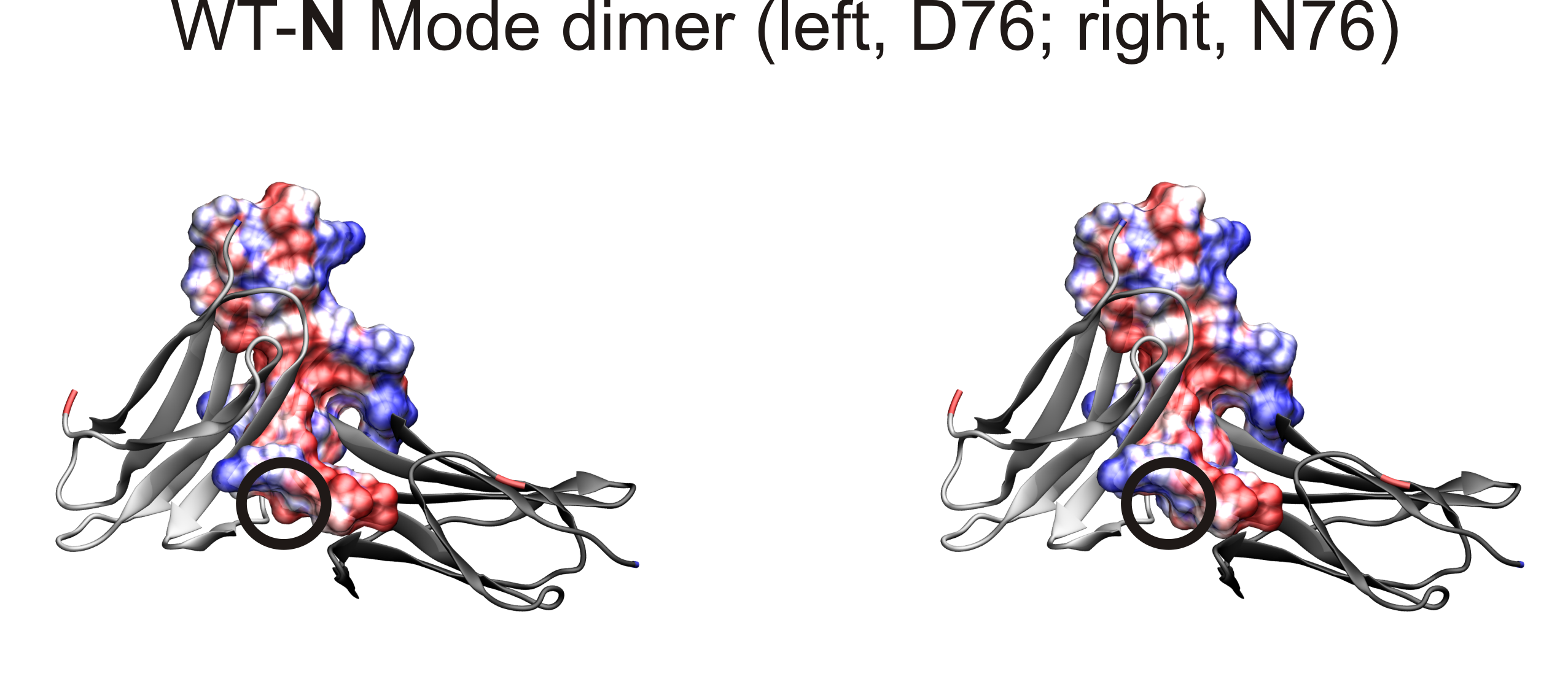

Supplement: Figure S4 — Surface electrostatic potentials of a typical (mode) WT-N dimer interface, with the original Asp76 (left) and the single-point mutation Asn76 (right), at physiological pH 7.2. The color transitions from red – white – blue when going from negative (−5 kBT/e) – neutral (0 kBT/e) – positive (+5 kBT/e) electrostatic potential. The regions around residue 76, located in the EF-loop, are circled. In the WT-N dimer interfaces the EF-loop of one monomer interacts, almost exclusively through Tyr78, with the second monomer’ DE-loop (Fig. S2A; Fig. S3A; Fig. 5A ). The dimer depicted is a representative example of such type of interaction. At physiological pH, the EF-loop of the WT-N has 3 negative charges and 1 positive charge. The DE loop displays 2 negative and 1 positive charges. In the WT-N dimer the interaction between both EF and DE loops can thus be affected by unfavorable electrostatic repulsions. The abundance of red color in the electrostatic map indicates a high load of negative charges in this type of interface (left) which is diminished when residue 76 is mutated into an Asn (right). The elimination of one negative charge from the EF-loop in the interface of the D76N dimer should therefore contribute to stabilize it, facilitating further oligomerization. The amino acids protonation states were attributed with PROPKA via the web server PDB2PQR v1.8 (http://nbcr-222.ucsd.edu/pdb2pqr_1.8/) and the calculation of the surface electrostatic potentials was done with the Adaptive Poisson-Boltzmann Solver – APBS v1.4 (http://www.poissonboltzmann.org/apbs/) and represented in VMD v1.8.7 (http://www.ks.uiuc.edu/Research/vmd/). (PNG) [file pcbi.1003606.s004.png]

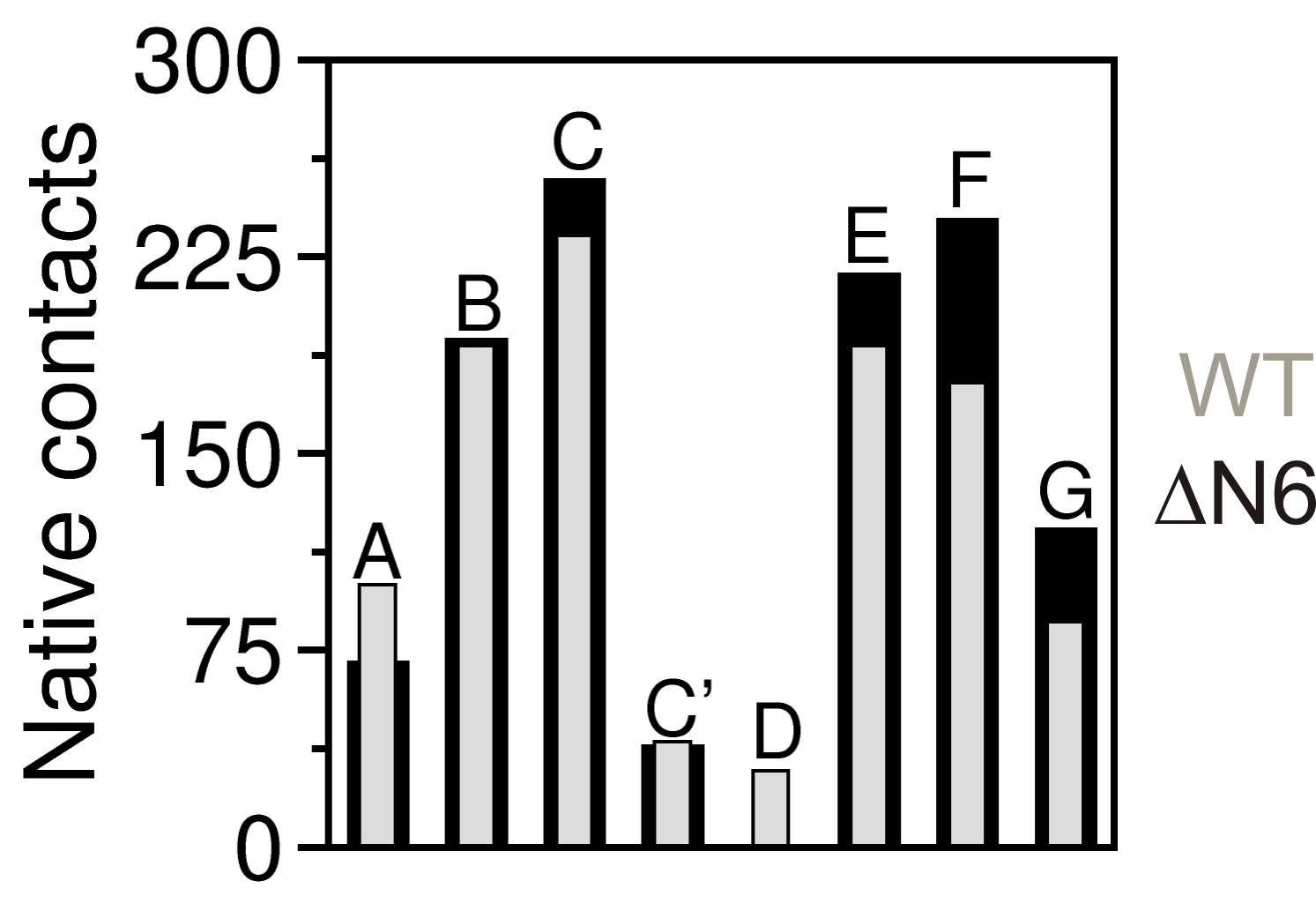

Supplement: Figure S5 — Native contacts in the Gō model. Number of native contacts per β-strand in the native structures of (WT) Hβ2m (PDB ID: 2XKS) and truncated variant ΔN6 (PDB ID: 2XKU). Secondary structure assignment is concurrent with the information provided in the PDB data files. In the WT form β-strands are defined within the sequence segments 6–11(A), 21–28(B), 36–41(C), 44–45(C′), 50–51(D), 64–70(E), 79–83(F), and 91–94(G). In the cleaved variant β-strands are defined in the following manner: 8–11(A), 21–27(B), 35–41(C), 44–45(C′), 64–70(E), 78–84(F), and 91–94(G). (PNG) [file pcbi.1003606.s005.png]
