# Supplementary material for: A Simulated Intermediate State for Folding and Aggregation Provides Insights into ΔN6 β2-Microglobulin Amyloidogenic Behavior
Source: PLoS Comput Biol. 2014 May 8;10(5):e1003606. doi: 10.1371/journal.pcbi.1003606 (PMC4014404; doi:10.1371/journal.pcbi.1003606)
Supplement: Table S1 — Structural characterization of the monomeric ΔN6-I sampled in the CpHMD simulations. The second column displays the mean Cα RMSD of the full chain fit to the native structure (PDB ID: 2XKU). The fifth column displays the SASA of the (βA+AB-loop) region. The remaining columns display the mean Cα RMSD of selected protein regions after fitting the core region, which comprises residues 21 to 94 (i.e. strands B–G and connecting loops), to the native structure. The RMSD of the (βA+AB-loop) region was obtained by taking into account the residues belonging to those structural elements plus the remaining N-terminus residues (residues 6–20). The BC region comprises residues 21–41 (strands B–C and BC-loop), the DE-region residues 50–70 (strands “D”–E and DE-loop) and the FG-region residues 78–94 (strands F–G and FG-loop). Averages were obtained from ensembles with ∼5000 conformations. (DOC) [file pcbi.1003606.s006.doc]

| **pH** | **Cα RMSD (Å)** | **Cα RMSD _21-94_ (Å)** | **β_A_ + AB-loop Cα RMSD (Å)** | **β_A_ + AB-loop SASA (Å^2^)** | **BC-region Cα RMSD (Å)** | **DE-region Cα RMSD (Å)** | **FG-region Cα RMSD (Å)** |
| --- | --- | --- | --- | --- | --- | --- | --- |
| 6.2 | 7.85 ± 0.95 | 5.23 ± 0.57 | 16.08 ± 2.98 | 1349 ± 117 | 3.72 ± 1.13 | 7.41 ± 0.83 | 2.44 ± 0.40 |
| 7.2 | 8.08 ± 1.11 | 6.72 ± 1.57 | 13.39 ± 1.52 | 1279 ± 129 | 5.95 ± 2.25 | 9.71 ± 2.21 | 3.05 ± 1.07 |
