# Supplementary material for: A Simulated Intermediate State for Folding and Aggregation Provides Insights into ΔN6 β2-Microglobulin Amyloidogenic Behavior
Source: PLoS Comput Biol. 2014 May 8;10(5):e1003606. doi: 10.1371/journal.pcbi.1003606 (PMC4014404; doi:10.1371/journal.pcbi.1003606)
Supplement: Table S2 — Structural characterization of the monomeric WT-N sampled in the CpHMD simulations. The second column reports the mean Cα RMSD of the full protein chain fit to the native structure (PDB ID: 2XKS). Values for the mean Cα RMSD21–94 were evaluated over the core region comprising residues 21 to 94 (i.e. strands B–G and connecting loops), after fitting to the core region of the native structure. Averages were obtained from ensembles with 2883 (6.2) and 3003 (7.2) conformations. (DOC) [file pcbi.1003606.s007.doc]

| **pH** | **Cα RMSD (Å)** | **Cα RMSD _21-94_ (Å)** |
| --- | --- | --- |
| 6.2 | 4.10 ± 1.09 | 2.18 ± 0.39 |
| 7.2 | 3.64 ± 1.48 | 2.11 ± 0.65 |
