# Supplementary material for: A Simulated Intermediate State for Folding and Aggregation Provides Insights into ΔN6 β2-Microglobulin Amyloidogenic Behavior
Source: PLoS Comput Biol. 2014 May 8;10(5):e1003606. doi: 10.1371/journal.pcbi.1003606 (PMC4014404; doi:10.1371/journal.pcbi.1003606)
Supplement: Table S3 — Structural characterization of the monomeric ΔN6-N sampled in the CpHMD simulations. The second column displays the average Cα RMSD of the full chain fit to the native structure (PDB ID: 2XKU). The remaining columns display the average Cα RMSD of selected protein regions after fitting the core region, which comprises residues 21 to 94 (i.e. strands B–G and connecting loops), to the native structure. The RMSD of the (βA+AB-loop) region was evaluated by taking into account the residues located within those structural elements plus the N-terminus residues (residues 6–20). The BC region comprises residues 21–41 (strands B–C and BC-loop), the DE-region residues 50–70 (strands “D”–E and DE-loop) and the FG-region residues 78–94 (strands F–G and FG-loop). Averages were obtained from ensembles with 1902(6.2) and 3003 (7.2) conformations. (DOC) [file pcbi.1003606.s008.doc]

| **pH** | **Cα RMSD (Å)** | **Cα RMSD _21-94_ (Å)** | **β_A_ + AB-loop Cα RMSD (Å)** | **BC-region Cα RMSD (Å)** | **DE-region Cα RMSD (Å)** | **FG-region Cα RMSD (Å)** |
| --- | --- | --- | --- | --- | --- | --- |
| 6.2 | 5.26 ± 1.08 | 4.14 ± 1.47 | 9.12 ± 1.67 | 2.18 ± 0.65 | 6.28 ± 2.42 | 1.82 ± 0.36 |
| 7.2 | 6.43 ± 1.70 | 5.53 ± 1.07 | 9.28 ± 5.59 | 4.77 ± 1.42 | 8.12 ± 1.57 | 2.54 ± 1.06 |
