# Supplementary material for: A Simulated Intermediate State for Folding and Aggregation Provides Insights into ΔN6 β2-Microglobulin Amyloidogenic Behavior
Source: PLoS Comput Biol. 2014 May 8;10(5):e1003606. doi: 10.1371/journal.pcbi.1003606 (PMC4014404; doi:10.1371/journal.pcbi.1003606)
Supplement: Table S4 — 50 most probable intermolecular contacts in ΔN6-I at pH 6.2. The color code is as follows: aromatic amino acids (i.e., Phe, Tyr, or Trp) interacting with Lys, Arg or His are highlighted in light blue. Aromatic amino acids interacting with an aromatic counterpart are highlighted in red (aromatic amino acids pairs can interact through their aromatic π rings in π–stacking interactions) while hydrophobic pairs (with an aromatic or aliphatic side chain) are highlighted in orange. Amino acids with electrically charged side chains (acidic – Asp and Glu; basic – Arg, Lys, and His) are highlighted in blue. Pairs involving an aromatic amino acid and His or Lys dominate the top 50 most frequent intermolecular contacts in ΔN6-I dimers at pH 6.2. These pairs can interact through cation-π interactions involving the aromatic π-ring and the positively charged moiety on Lys or protonated His (when neutral, His can establish aromatic–aromatic or π–stacking interactions with its aromatic partner as well as δ+-π interactions due to polarization). Arg and Lys side chains are protonated (positively charged) in the range of pHs studied (pK a>>pH). Glu and Asp RCOO− side chains are mostly unprotonated (negatively charged) in physiological or near-physiological pHs. His imidazole ring can become protonated in the 6.2–7.2 pH range. Assuming an average pK a of the imidazole ring side chain of approximately 6.5, only 17% of all His in the system become protonated at a pH of 7.2 while at a slightly lower pH of 6.2 this value increases up to 67% (the side chain pK a of a buried His can assume a value under 6.5 thus lowering the pH for which it becomes protonated). (DOC) [file pcbi.1003606.s009.doc]

| Residue ID  (1) | Residue ID  (2) | Residue Name  (1) | Residue Name  (2) |
| --- | --- | --- | --- |
| 31 | 60 | HIS | TRP |
| 60 | 74 | TRP | GLU |
| 60 | 10 | TRP | TYR |
| 84 | 10 | HIS | TYR |
| 60 | 16 | TRP | GLU |
| 60 | 8 | TRP | GLN |
| 13 | 75 | HIS | LYS |
| 60 | 84 | TRP | HIS |
| 17 | 60 | ASN | TRP |
| 30 | 75 | PHE | LYS |
| 10 | 84 | TYR | HIS |
| 75 | 30 | LYS | PHE |
| 74 | 10 | GLU | TYR |
| 59 | 10 | ASP | TYR |
| 75 | 10 | LYS | TYR |
| 87 | 30 | LEU | PHE |
| 60 | 60 | TRP | TRP |
| 30 | 84 | PHE | HIS |
| 10 | 89 | TYR | GLN |
| 30 | 60 | PHE | TRP |
| 84 | 84 | HIS | HIS |
| 56 | 13 | PHE | HIS |
| 13 | 84 | HIS | HIS |
| 10 | 87 | TYR | LEU |
| 60 | 31 | TRP | HIS |
| 60 | 59 | TRP | ASP |
| 30 | 9 | PHE | VAL |
| 60 | 13 | TRP | HIS |
| 60 | 73 | TRP | THR |
| 89 | 10 | GLN | TYR |
| 10 | 10 | TYR | TYR |
| 13 | 30 | HIS | PHE |
| 60 | 7 | TRP | ILE |
| 30 | 85 | PHE | VAL |
| 8 | 84 | GLN | HIS |
| 87 | 10 | LEU | TYR |
| 74 | 30 | GLU | PHE |
| 87 | 60 | LEU | TRP |
| 30 | 87 | PHE | LEU |
| 75 | 31 | LYS | HIS |
| 31 | 10 | HIS | TYR |
| 60 | 75 | TRP | LYS |
| 16 | 60 | GLU | TRP |
| 17 | 63 | ASN | TYR |
| 87 | 84 | LEU | HIS |
| 63 | 60 | TYR | TRP |
| 16 | 78 | GLU | TYR |
| 17 | 81 | ASN | ARG |
| 74 | 60 | GLU | TRP |
| 89 | 30 | GLN | PHE |
